# Scientometric trends for coronaviruses and other emerging viral infections

**DOI:** 10.1093/gigascience/giaa085

**Published:** 2020-08-17

**Authors:** Dima Kagan, Jacob Moran-Gilad, Michael Fire

**Affiliations:** Department of Software and Information Systems Engineering, Ben-Gurion University of the Negev, P.O.B 653, 8410501, Beersheba, Israel; Department of Health Systems Management, Faculty of Health Sciences, Ben-Gurion University of the Negev, P.O.B 653, 8410501, Beersheba, Israel; Department of Software and Information Systems Engineering, Ben-Gurion University of the Negev, P.O.B 653, 8410501, Beersheba, Israel

**Keywords:** coronavirus, emerging viruses, epidemics, SARS

## Abstract

**Background:**

COVID-19 is the most rapidly expanding coronavirus outbreak in the past 2 decades. To provide a swift response to a novel outbreak, prior knowledge from similar outbreaks is essential.

**Results:**

Here, we study the volume of research conducted on previous coronavirus outbreaks, specifically SARS and MERS, relative to other infectious diseases by analyzing >35 million articles from the past 20 years. Our results demonstrate that previous coronavirus outbreaks have been understudied compared with other viruses. We also show that the research volume of emerging infectious diseases is very high after an outbreak and decreases drastically upon the containment of the disease. This can yield inadequate research and limited investment in gaining a full understanding of novel coronavirus management and prevention.

**Conclusions:**

Independent of the outcome of the current COVID-19 outbreak, we believe that measures should be taken to encourage sustained research in the field.

## Introduction

Infectious diseases remain a major cause of morbidity and mortality worldwide, in developed countries and particularly in the developing world [1]. According to the World Health Organization (WHO), among the top 10 causes of death globally, 3 are infectious diseases [1]. In light of the continuous emergence of infections, the burden of infectious diseases is expected to become even greater in the near future [2, 3]. Many emerging pathogens are RNA viruses, and notable examples over the past 2 decades include the SARS coronavirus in 2002–2003 in China, pandemic influenza (swine flu) A/H1N1 in 2009, the MERS coronavirus in 2012 in the Middle East, and Ebola virus disease in 2013–2014 in Africa.

Currently, the world is struggling with a novel strain of coronavirus (SARS-CoV-2) that emerged in China during late 2019 and by the time of this writing has infected >19,500,000 people and killed >700,000 [4,5]. COVID-19 is the latest and third serious human coronavirus outbreak in the past 20 years. Additionally, of course, there are several more typical circulating seasonal human coronaviruses causing respiratory infections. COVID-19, is already a pandemic that that is more difficult to contain than its close relative SARS-CoV [6,7].

Much can be learned from past infectious disease outbreaks to improve preparedness and response to future public health threats. Three key questions arise in light of the COVID-19 outbreak: To what extent were the previous human coronavirus (SARS and MERS) outbreaks studied? Is research on emerging viruses being sustained, aiming to understand and prevent future epidemics? Are there lessons from academic publications on previous emerging viruses that could be applied to the current COVID-19 epidemic?

In this study, we answer these vital questions by utilizing state-of-the-art data science tools to perform a large-scale analysis of 35 million papers, of which 1,908,211 concern the field of virology. We explore nearly 2 decades of infectious disease research published from 2002 up to today. We particularly focus on public health crises, such as SARS, influenza (including seasonal, pandemic H1N1, and avian influenza), MERS, and Ebola virus disease, and compare them to HIV/AIDS and viral hepatitis B and C, 3 bloodborne viruses that are associated with a significant global health burden for >2 decades.

A crucial aspect of being prepared for future epidemics is sustained ongoing research of emerging infectious diseases even at “times of peace" when such viruses do not pose an active threat. Our results demonstrate that research on previous coronaviruses, such as SARS and MERS, was conducted by a relatively small number of researchers centered in a small number of countries, suggesting that such research could be better encouraged. We propose that regardless of the fate of COVID-19 in the near future, sustained research efforts should be encouraged to better prepare for the next outbreak.

## Background

This research is a large-scale scientometric study in the field of infectious diseases. We focus on the quantitative features and characteristics of infectious disease research over the past 2 decades. In this section, we present studies that analyze and survey real-world trends in the field of infectious diseases (see the Infectious Disease Trends subsection) and studies that relate to bibliometric trends in general and public health in particular (see the Bibliometric Trends subsection).

### Infectious disease trends

There is great promise in utilizing big data to study epidemiology [8]. One approach is to gather data using different surveillance systems. For example, one such system is ProMED. ProMED was launched 25 years ago as an email service to identify unusual worldwide health events related to emerging and reemerging infectious diseases [9]. It is used daily around the globe by public health policy makers, physicians, veterinarians, and other health care workers, researchers, private companies, journalists, and the general public. Reports are produced and commentary is provided by a global team of subject matter experts in a variety of fields. ProMED has >80,000 subscribers and >60,000 cumulative event reports from almost every country in the world. Additionally, there are many different systems used by different countries and health organizations worldwide.

In 2006, Cowen et al. [10] evaluated the ProMED dataset from the years 1996 to 2004. They discovered that there are diseases that received more extensive coverage than others: “86 disease subjects had thread lengths of at least 10 reports, and 24 had 20 or more.” They note that the pattern of occurrence is hard to explain even by an expert in epidemiology. Also, with the level of granularity of ProMED data, it is very challenging to predict the frequency with which diseases are going to accrue. In 2008, Jones et al. [2] analyzed the global temporal and spatial patterns of emerging infectious diseases (EIDs). They analyzed 305 EIDs between 1940 and 2004 and demonstrated that the threat of EIDs to global health is increasing. The same year, Freifeld et al. [11] developed HealthMap, an interactive surveillance system that integrates disease outbreak reports from various sources.

Data about infectious diseases can also come from web- and social-based sources. For instance, in 2009, Ginsberg et al. [12] used Google search queries to monitor the spread of influenza epidemics. They used the fact that many people search online before consulting physicians, and they found that during a pandemic, the volume of searches differs from normal. They then created a mathematical model to forecast the spread of influenza. This research was later converted into a tool called Google Flu Trends, and at its peak, Google Flu Trends was deployed in 29 countries worldwide. However, not everything worked well for Google Flu Trends; in 2009, it underestimated the flu volume, and in 2013, it predicted more than double the number of cases compared with the true volume [13]. As a result of such discrepancies, Google shut down the Google Flu Trends website in 2015 and transferred its data to academic researchers [14]. Also in 2009, Carneiro and Mylonakis [15] used large amounts of data to predict influenza outbreaks a week earlier than prevention surveillance systems.

In 2010, Lampos and Cristianini [16] extended the idea of Carneiro and Mylonakis [15] to use temporal data to monitor outbreaks. Instead of using Google Trends, they used Twitter as their data source. They collected 160,000 tweets from the UK, and as ground truth, they used Health Protection Agency weekly reports about the H1N1 epidemic. Using textual markers to measure influenza on Twitter, they demonstrated that Twitter can be used to study disease outbreaks, similar to Google Trends. In 2011, Salathé and Khandelwal [17] analyzed Twitter and demonstrated that it is possible to use social networks to study not only the spread of infectious disease but also vaccinations. They found a correlation between the sentiment in tweets toward an influenza vaccine and the vaccination rate.

In 2014, Generous et al. [18] used Wikipedia to monitor and forecast infectious disease outbreaks. They examined Wikipedia access logs to forecast outbreak volumes for 14 combinations of diseases and locations. The model worked successfully for only 8 of the 14 cases. Also, the authors suggested that it was even possible to transfer a model between locations without retraining it. In contrast to most of the web-based disease-monitoring methods, Wikipedia-based monitoring presents a fully open forecasting system that can be easily reproducible. Generally, in the past couple of years, Wikipedia has become a widely used data source for medical studies [19,20]. Moreover, a recent report [21] shows that Wikipedia has successfully kept itself free of the misinformation spread during the COVID-19 outbreak. In 2015, Santillana et al. [22] took the influenza surveillance one step further by fusing multiple data sources. They used 5 datasets: Twitter, Google Trends, near real-time hospital visit records, FluNearYou, and Google Flu Trends. They used all these data sources with a machine-learning algorithm to predict influenza outbreaks. In 2017, McGough et al. [23] dealt with the problem of significant delays in the publication of official government reports about Zika cases. To solve this problem, they used the combined data of Google Trends, Twitter, and the HealthMap surveillance system to predict estimates of Zika cases in Latin America.

In 2018, Breugelmans et al. [24] explored the effects of publishing in open access journals and collaboration between European and sub-Saharan African researchers in the study of poverty-related disease. To this end they used the PubMed dataset but discovered that it is not suited to performing full bibliometric analysis; to deal with this issue they also utilized Web of Science as a data source. They discovered that there is an advantage for open access publications in terms of citations. In 2020, Head et al. [25] studied infectious disease funding. They discovered that HIV/AIDS is the most funded disease. Additionally, they discovered a pattern according to which Ebola, Zika, influenza, and coronavirus funding were highest after an outbreak.

There is substantial controversy surrounding the use of web-based data to predict the volume of outbreaks. The limitations of Google Flu Trends raised the question of reliability of social data for assessing disease spread. Lazer et al. [26] noted that these types of methods are problematic because companies like Google, Facebook, and Twitter are constantly changing their products. Studies based on such data sources may be valid today but not tomorrow, and may even be unreproducible.

### Bibliometric trends

In 2005, Vergidis et al. [27] used PubMed and JCR (Journal Citation Reports) to study trends in microbiology publications. They discovered that microbiology research in the USA had the highest average impact factor, but in terms of research production, Western Europe was first. In 2008, Uthman [28] analyzed trends in paper publications about HIV in Nigeria. He found growth (from 1 to 33) of the number of publications about HIV in Nigeria and that papers with international collaborations were published in journals with a higher impact factor. In 2009, Ramos et al. [29] used Web of Science to study publications about infectious diseases in European countries. They found that more papers in total were published about infectious diseases in Europe than in the USA.

In 2012, Takahashi-Omoe and Omoe [30] surveyed publications of 100 journals about infectious diseases. They discovered that the USA and the UK had the highest number of publications, and relative to the country's socioeconomic status, the Netherlands, India, and China had relatively high productivity. In 2014, similar to Wislar et al. [31], Kennedy et al. [32] studied ghost authorship in nursing journals instead of biomedical journals. They found that there were 28% and 42% of ghost and honorary authorships, respectively.

In 2015, Wiethoelter et al. [33] explored worldwide infectious disease trends at the wildlife-livestock interface. They found that 7 of the top 10 most studied diseases were zoonoses. In 2017, Dong et al. [34] studied the evolution of scientific publications by analyzing 89 million papers from the Microsoft Academic dataset. Similar to the increase found by Aboukhalil [35], they also found a drastic increase in the number of authors per paper. In 2019, Fire and Guestrin [36] studied the over-optimization in academic publications. They found that the number of publications has ceased to be a good metric for academic success as a result of longer author lists, shorter papers, and surging publication numbers. Citation-based metrics, such as citation number and h-index, are likewise affected by the flood of papers, self-citations, and lengthy reference lists.

## Data Description

In this study, we fused 4 data sources to extract insights about research on emerging viruses. In the rest of this subsection we describe these data sources.

1. MAG—Microsoft Academic Graph is a dataset containing “scientific publication records, citation relationships between those publications, as well as authors, institutions, journals, conferences, and fields of study” [37]. The MAG dataset we used was from 22 March 2019 and contains data on >210 million papers [38]. This dataset was used as the main dataset of the study. Similar to Fire and Guestrin [36], we only used papers that had ≥5 references in order to filter out non peer-reviewed publications, such as news columns that are published in journals.
2. PubMed—PubMed is a dataset based on the PubMed search engine of academic publications on the topics of medicine, nursing, dentistry, veterinary medicine, health care systems, and preclinical sciences [39]. One of the major advantages of using the PubMed dataset is that it contains only medical-related publications. The data on each PubMed paper contain information about its venue, authors, and affiliations, but they do not contain citation data. In this study, we used the 2018 annual baseline PubMed dataset containing 29,138,919 records [40]. We mainly utilized the PubMed dataset to analyze journal publications (see Paper Trends section).
3. SJR—Scientific Journal Rankings is a dataset containing the information and ranking of >34,100 journals from 1999 to 2018 [41], including their SJR indicator (a measure used to assess the prestige of a journal, SJR takes into account the number of citations and the prestige of the source of the citing paper [42]), the best quartile of the journal (“The Journal Impact Factor quartile is the quotient of a journal's rank in category [X] and the total number of journals in the category [Y], so that [X / Y] = Percentile Rank Z” [43]), and more. We utilized the SJR dataset to compare the rankings of different journals to assess the level of their prestige.
4. Wikidata—Wikidata is a dataset holding a vast knowledge about the world, containing data on >78,252,808 items [44]. Wikidata stores metadata about items, and each item has an identifier and can be associated with other items. We utilized the Wikidata dataset to extract geographic information for academic institutions in order to match a paper with its authors' geographic locations.

## Analyses

### Infectious disease analysis

To study the research of emerging viruses over time, we analyzed the datasets described in the Data Description section. In pursuing this goal, we used the code framework recently published by Fire and Guestrin [36], which enables the easy extraction of the structured data of papers from the MAG dataset. The MAG and PubMed datasets were filtered according to a predefined list of keywords. The keyword search was performed in the following way: given a set of diseases *D* and a set of papers *P*, from each paper title *p_t_*, where *p* ∈ *P*, we created a set of “word-grams." Word-grams are defined as *n-*grams of words, i.e., all the combinations of a set of words in a phrase, without disrupting the order of the words. For example, the word-gram*s* of the string “Information on Swine Flu,” word-grams(InformationonSwineFlu), will return the following set: {Information,on,Swine,Flu,Information on,on Swine,Swine Flu,Information on Swine,on Swine Flu,Information on Swine Flu}. Next, for each *p*, we calculated word-gram(*p_t_*) ∩ *D*, which was considered as the diseases with which the paper was associated.

In the present study, we focused on the past emerging coronaviruses (SARS and MERS). There are many other strains of the human coronavirus, and 4 of them are known for causing seasonal respiratory infections [45]. We focused on SARS and MERS because they are more closely related to SARS-CoV-2 and both have zoonotic origins and raised international public health concern. Additionally, we also analyzed Ebola virus disease, influenza (seasonal, avian influenza, swine flu), HIV/AIDS, hepatitis B, and hepatitis C as comparators that represent other important emerging infectious diseases from the past 2 decades. For these 9 diseases, we collected all their aliases, which were added to the set of diseases *D* and were used as keywords to filter the datasets. To reduce the false-positive rate, we analyzed only papers that, according to the MAG dataset, were in the categories of medicine or biology, and following Fire and Guestrin [36] had ≥5 references. Additionally, to explore the trend in the core categories of infectious disease research, we performed the same analysis on the virology category. In the rest of this section, we describe the specific calculations and analyses we performed.

#### Paper trends

To explore the volume of studies on emerging viruses, we examined the publication of papers about infectious diseases. First, we defined several notions that we used to define publication and citation rates. Let *D* be a set of disease names and *P* a set of papers. Namely, for a paper *p* ∈ *P, p*_Disease_ is defined as the disease that matches the paper's keywords, *p*_Year_ as the paper's publication year, and *p*_citations_ as the set of papers citing *p*. Using these notions, we defined the following features:

- Number of Citations—the total number of citations for a specific infectious disease.
- Number of Papers—the total number of published papers for a specific infectious disease.
- Normalized Citation Rate (NCR_*y*_)—the ratio between the Number of Citations on a specific infectious disease *d* and the total number of citations about medicine or biology in year *y* (to determine which papers, we used the MAG fields of study).
  (1) \begin{equation*}
\mathrm{NCR}_{y}(d) = \frac{\sum _{\lbrace i \in P | p_{\mathrm{Year}}=y \mbox{ and } i_{\mathrm{Disease}}=d\rbrace }\sum _{\lbrace j \in P \rbrace } j \in i_{\mathrm{citations}}}{|\lbrace j \in P | j_{\mathrm{Year}}=y\rbrace j_{\mathrm{citations}}|}
\end{equation*}
- Normalized Paper Rate (NPR)—the ratio between the Number of Papers published on a specific infectious disease *d* and the total number of papers in the fields of medicine or biology in the year *y*.
  (2) \begin{equation*}
\mathrm{NPR}_{y}(d) = \frac{|\lbrace i \in P | i_{\mathrm{Year}}=y \mbox{ and } i_{\mathrm{Disease}}=d\rbrace |}{|\lbrace i \in P | i_{\mathrm{Year}}=y\rbrace |}
\end{equation*}

Using these metrics, we inspected how the coronavirus publication and citation rates differed from those of other examined EIDs. We analyzed how trends of citations and publications have changed over time. Additionally, to inspect the similarities between the trends of different diseases we calculated the dynamic time warping (DTW) distance [46] between all the disease pairs. Finally, we clustered the time series using TimeSeriesKMeans [47].

#### Journal trends

To investigate the relationship between journals and their publication of papers about emerging viruses, we combined the Semantic Scholar and PubMed datasets with the SJR dataset using ISSN, and selected all the journals from SJR categories related to infectious diseases (immunology, epidemiology, infectious diseases, virology, and microbiology). First, we inspected whether coronavirus papers are published in the top journals. We selected the top 10 journals by SJR and calculated the number of papers they had published for each disease over time. Next, we inspected how published papers about coronavirus are regarded relative to those about other EIDs in terms of ranking. To this end, we defined a new metric, JScore_*t*_. JScore_*t*_ is defined as the mean SJR score of all published papers on a specific topic *t*. We used JScore_*t*_ to observe how the prominence of each disease in the publication world has changed over time. Last, we explored publications by looking at the quartile ranking of the journal over time.

#### Author trends

To study how scientific authorship has changed in the field of infectious diseases, we explored what characterizes the authors of papers on different diseases. We inspected the number of new authors over time to check how attractive emerging viruses are to new researchers. Additionally, we analyzed the authors experience, where author experience is defined as the time that has passed from his or her first publication. The authors were identified by the identification number provided in the MAG dataset. Author disambiguation is a challenging task; Microsoft combined multiple methods to generate their author identifications [48]. We also analyzed the number of authors who wrote multiple papers about each disease.

#### Collaboration trends

To inspect the state of international collaborations in emerging virus research, we mapped academic institutions to geolocation. However, it is not a trivial task to match institution names. Institution names are sometimes written differently; e.g., Aalborg University Hospital and Aalborg University are affiliated. However, there are cases where 2 similar names refer to different institutions; e.g., the University of Washington and Washington University are entirely different institutions. To deal with this problem, we used the affiliation table in the MAG dataset. To determine the country and city of each author, we applied a 5-step process:

1. For each institution, we looked for the institution's page on Wikidata. From each Wikidata page, we extracted all geography-related fields (the fields used were “coordinate location (P625),” “country (P17),” “located at street address (P6375),” “located in the administrative territorial entity (P131),” “headquarters location (P159),” and “location (P276).”).
2. To first merge all the Wikidata location fields, we used the “coordinate location” with reverse geocoding to determine the city and country of the institution.
3. For all the institutions that did not have a “coordinate location” field, we extracted the location data from the other available fields. We crossed the data against city and country lists from GeonamesCache Python library [49] to determine whether the data in the field described a city or a country.
4. To acquire country data for an institution that had only city data on Wikidata, we used GeonamesCache city-to-country mapping lists.
5. To get city and country data for institutions that did not have the relevant fields on Wikidata, we extracted geographic coordinates from Wikipedia.org (English Wikipedia). Even though Wikidata and Wikipedia.org are both operated by the Wikimedia Foundation, they are independent projects that have different data. Similar to Wikidata coordinates, we used reverse geocoding to determine the city and country of the institution.

Using the extracted geodata, we explored how international collaborations change over time in coronavirus research. Finally, we explored which countries have the highest number of papers about coronavirus and which countries have the highest number of international collaborations over time.

## Results

In the following subsections, we present all the results of the experiments that were described in the Analyses section.

### Results of paper trends

**Figure 1:**
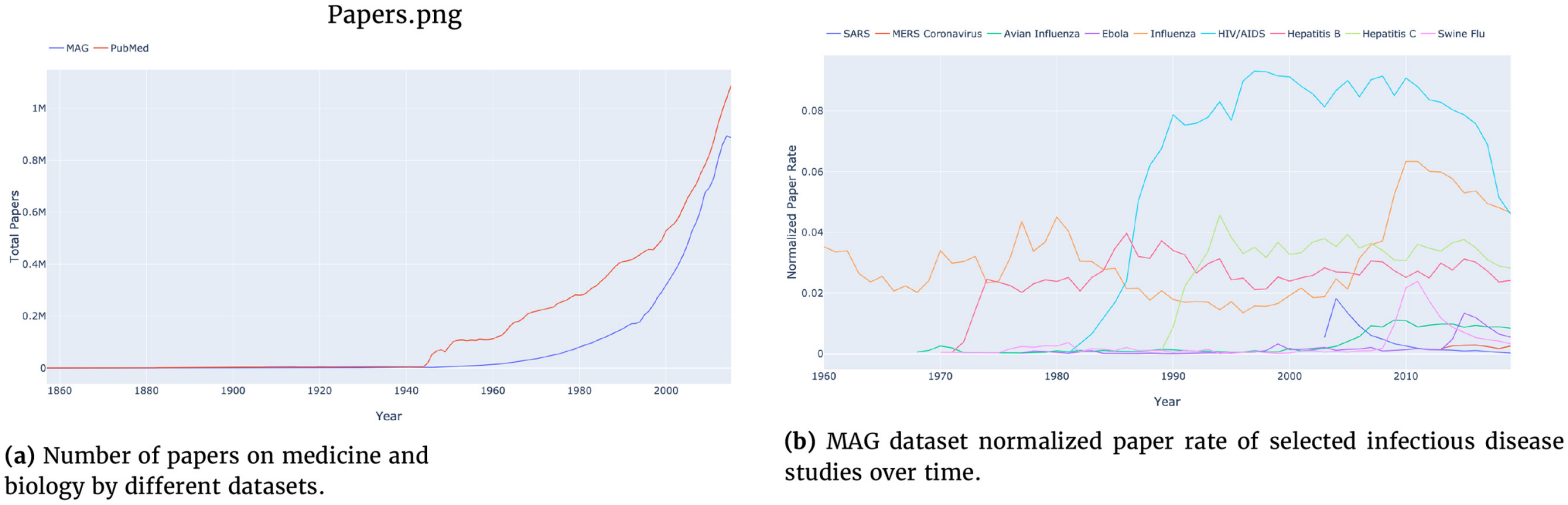
The number of papers over time.

**Figure 2:**
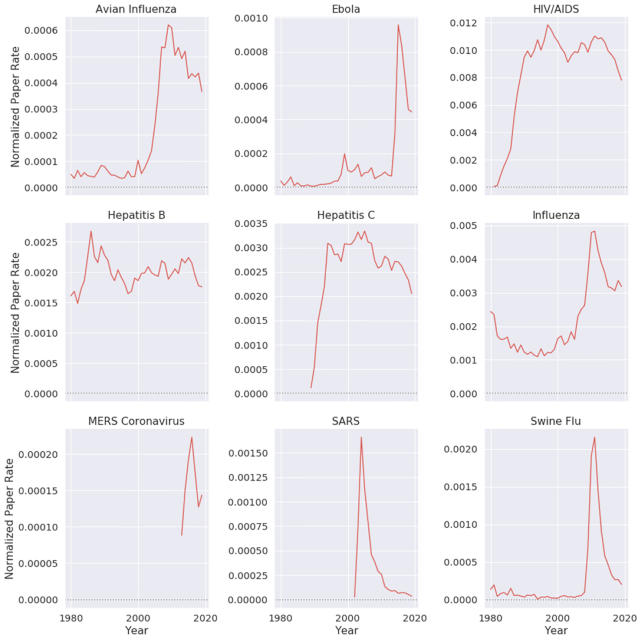
Normalized paper rate by different diseases over time. Diseases that have a drastic increase in their normalized number of publications mostly coincide with an epidemic.

**Figure 3:**
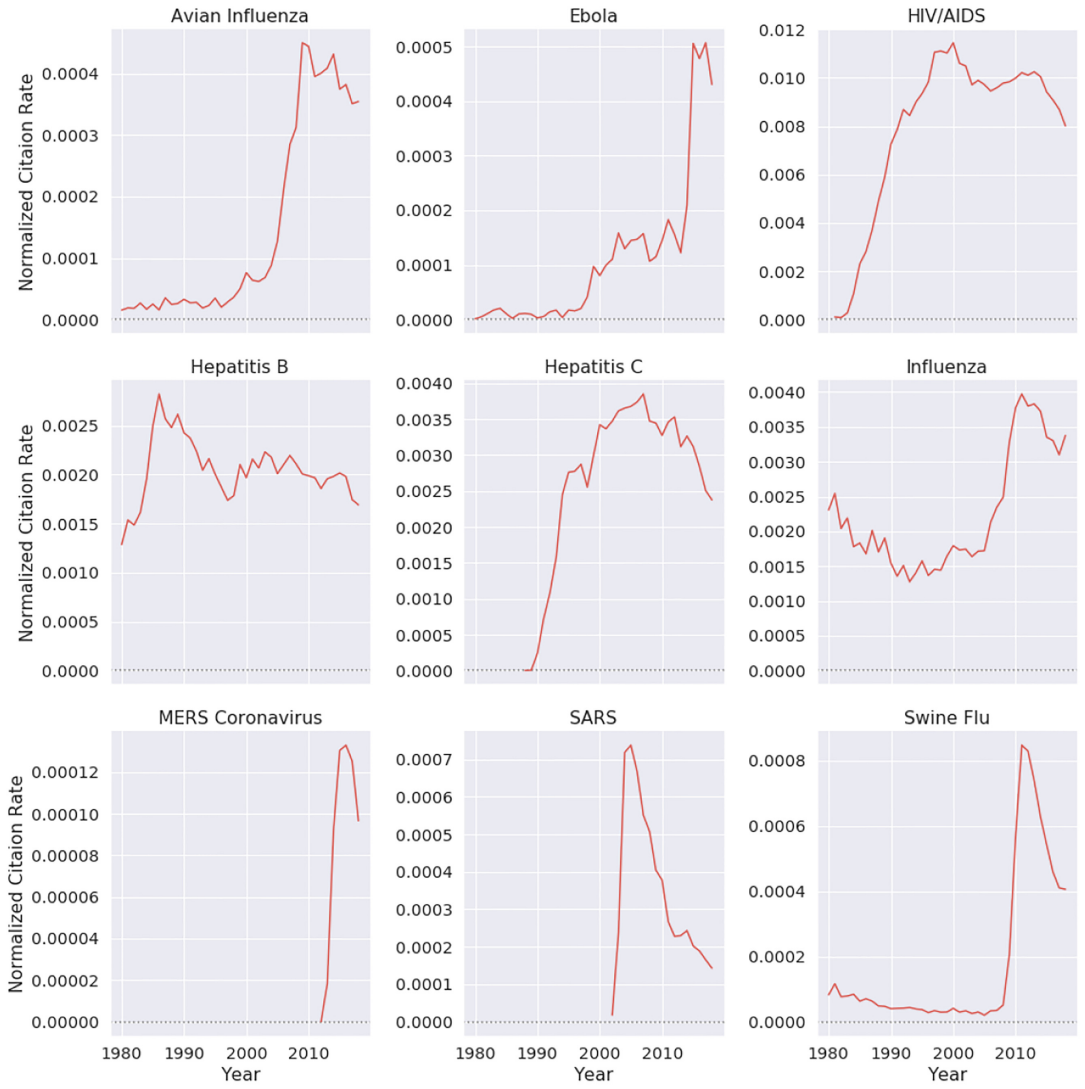
Normalized citation rate by different diseases over time. Diseases that have a drastic increase in their normalized number of citations mostly represent an outbreak.

In recent years, there has been a surge in academic publications, yielding >1 million new papers related to medicine and biology each year (see Fig. 1a). In contrast to the overall increase in the number of infectious disease papers, there has been a relative decline in the number of papers about the coronaviruses SARS and MERS (see Fig. 1b). Also, we found that 0.4% of virology studies in our corpus from the past 20 years involved human SARS and MERS, while HIV/AIDS accounts for 7.9% of all virology studies. We observed that, unlike the research in the domain of HIV/AIDS and avian influenza that has been published at a high and steady pace over the past 20 years, SARS was studied at an overwhelming rate after the 2002–2004 outbreak and then sharply decreased after 2005 (Fig. 2). In terms of Normalized Paper Rate (see Fig. 2), after the first SARS outbreak, there was a peak in publishing SARS-related papers with NPR twice as high as Ebola's. However, the trend decreased very quickly, and a similar phenomenon can be observed for the swine flu pandemic. The MERS outbreak achieved a much lower NPR than SARS, specifically >16 times lower when comparing the peaks in SARS and MERS trends. In terms of Normalized Citation Rate (Fig. 3), we observed the same phenomenon as we did with NPR. Observing Figs 9 and 10, we can see that there are diseases with very similar trends. More precisely, NPR and NCR trends are in 2 clusters, where the first cluster contains avian influenza, Ebola, MERS, SARS, and swine flu and the second cluster contains HIV/AIDS, hepatitis B, hepatitis C, and influenza.

### Results of journal trends

**Figure 4:**
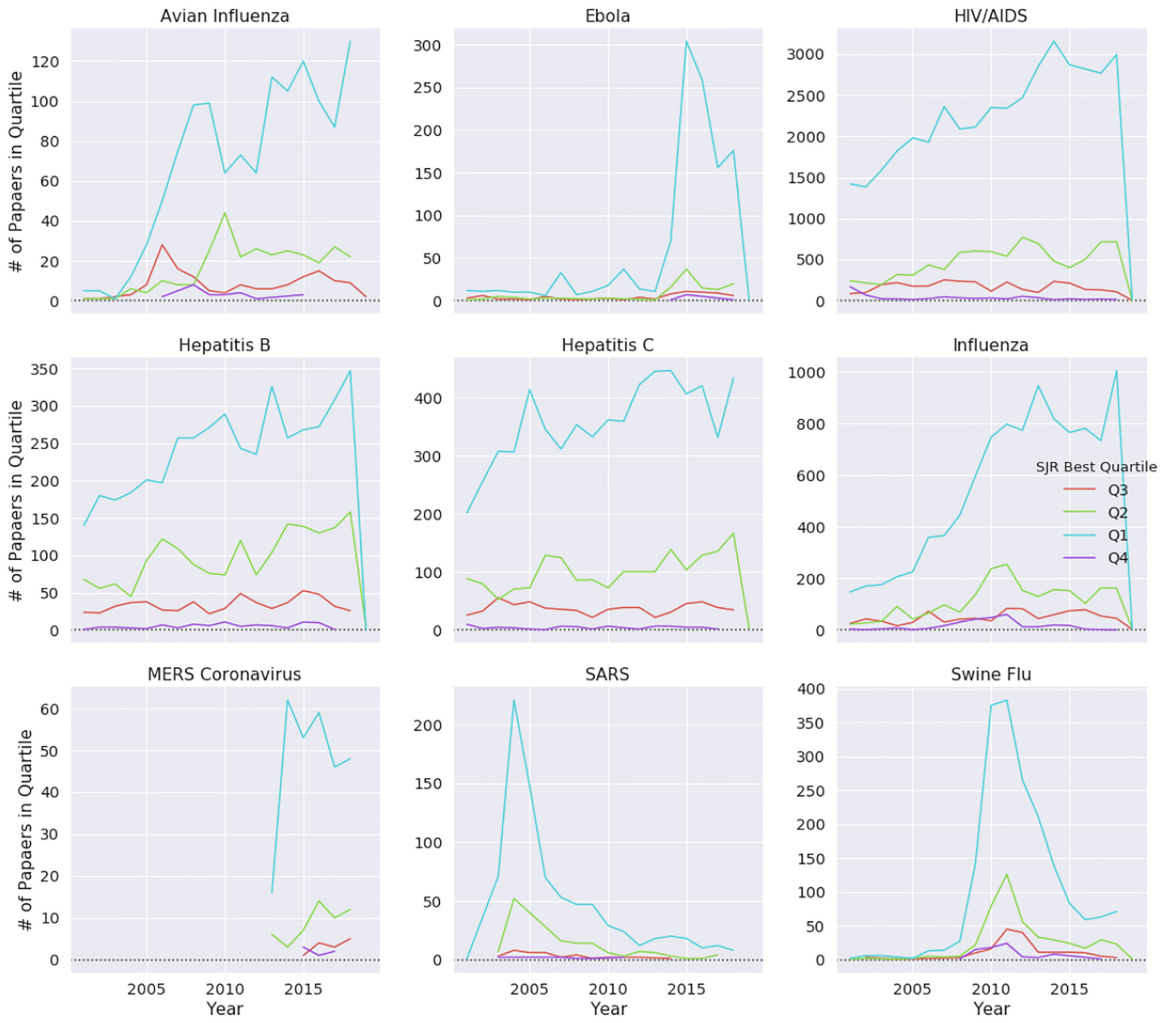
Publications by quartile over time for different diseases. Unlike other emerging infectious diseases, avian influenza did not demonstrate a decline in Q1 publications.

**Figure 5:**
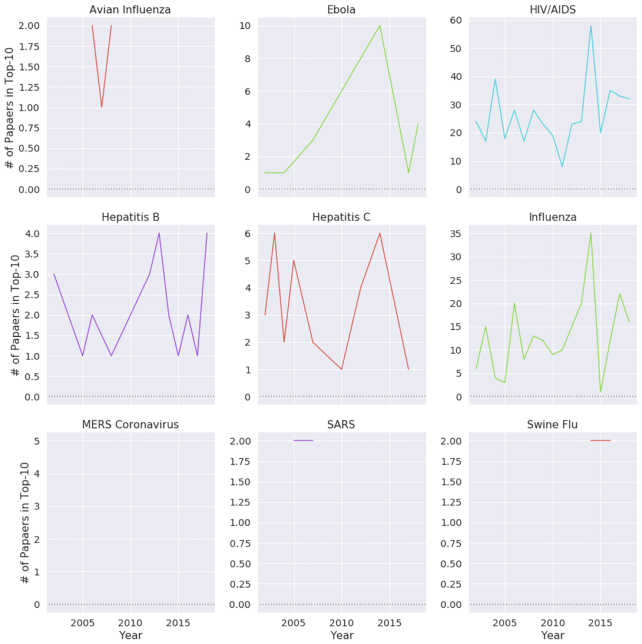
Number of papers by top-10 publications over time for different diseases.

**Figure 6:**
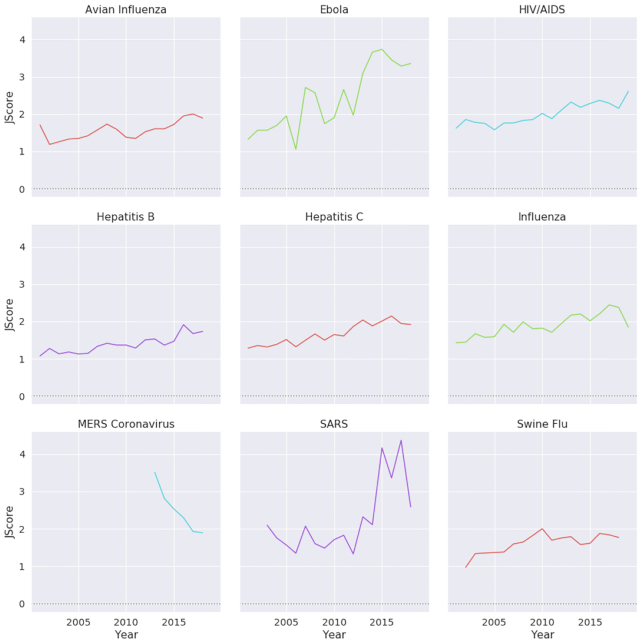
JScore over time for different diseases. Except for MERS, all presented diseases show an increase in JScore.

From analyzing the trends in journal publications, we discovered that the numbers of papers published by journal quartile are very similar to Normalized Paper Rate and Normalized Citation Rate (see Fig. 4). We observed that for most of the diseases, the trends are quite similar: an increase in the study rate is coupled with a greater number of published papers in Quartile 1 (Q1) journals. We discovered that for SARS, MERS, the swine flu, and Ebola, Q1 publication trends were almost parallel to their NPR trends (see Figs 2 and 4). Also, we noticed that HIV, avian influenza, influenza, and hepatitis B and C have steady publication numbers in Q1 journals. Looking at papers in highly ranked journals (Fig. 5), we observed that the diseases that are being continuously published in top-10–ranked journals are mainly persisting diseases, such as HIV and influenza. Additionally, we inspected how the mean journal ranking of publications by disease has changed over time (Fig. 6). We found that only MERS had a decline of JScore. We also noticed that current papers about SARS had the highest JScore.

### Results of author trends

By studying the authorship trends in the research of emerging viruses, we discovered that there is a difference in the mean experience of authors among diseases. SARS researchers had the lowest experience in years, and hepatitis C had the most experienced researchers (see Table 1). We noticed that the SARS research community had a smaller percentage of relatively prolific researchers than other diseases. Moreover, researchers with multiple papers related to SARS and MERS published on average 3.84 and 3.86 papers, respectively, while hepatitis C researchers published on average 5.24 papers during the same period. Additionally, from analyzing authors who published multiple papers on a specific disease, we found that on average there was a 2.5-paper difference between HIV and SARS authors. Furthermore, swine flu, SARS, and MERS were the diseases on which authors published the fewest multiple papers.

**Table 1:** Median researcher experience in years by disease

Disease	Median experience in years
SARS	4
Avian influenza	5
Swine flu	5
Hepatitis B	5
Ebola	5
Influenza	6
HIV/AIDS	7
MERS Coronavirus	7
Hepatitis C	8

### Results of collaboration trends

**Figure 7:**
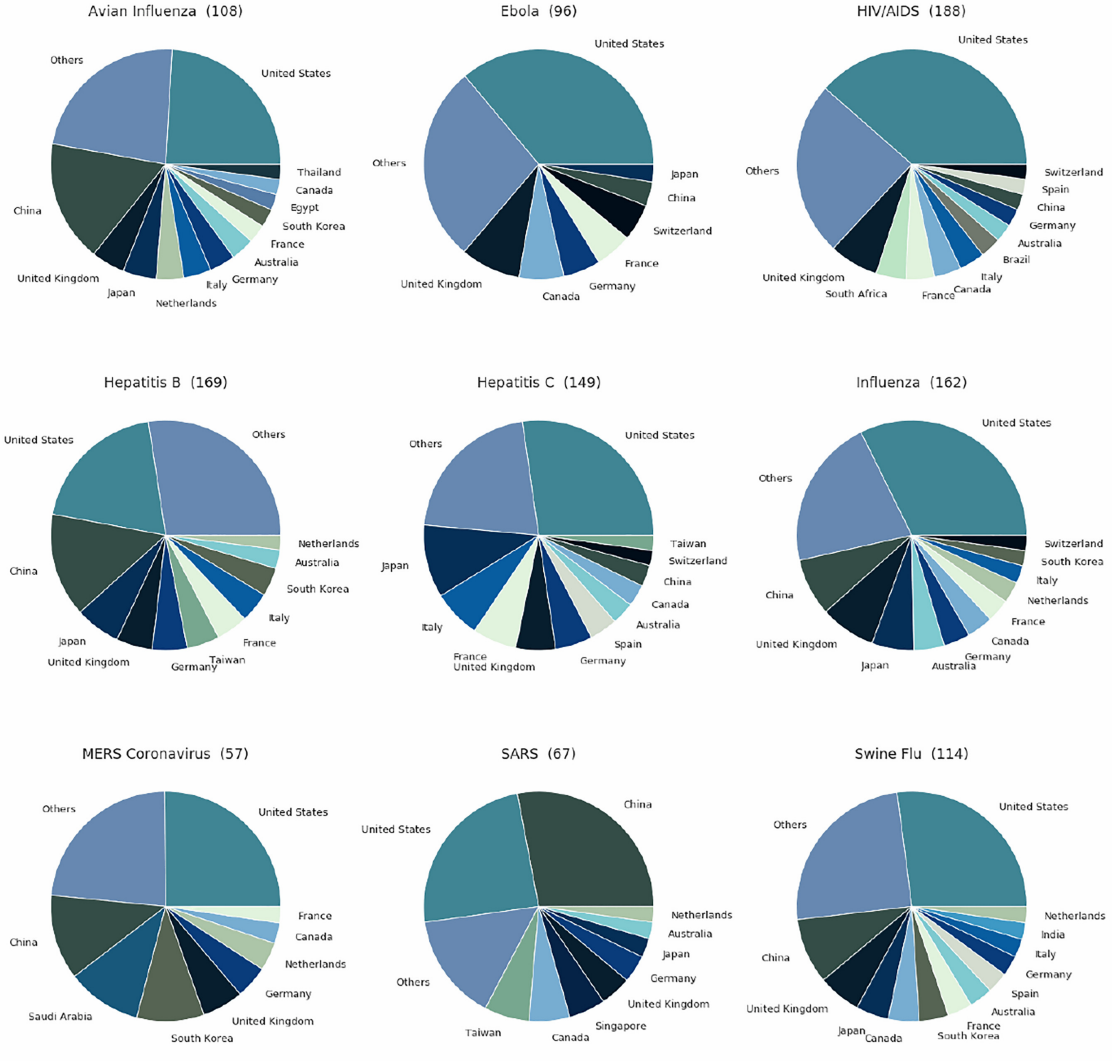
Number of researchers in each country for each disease. Most of the research was conducted in a small number of countries.

**Figure 8:**
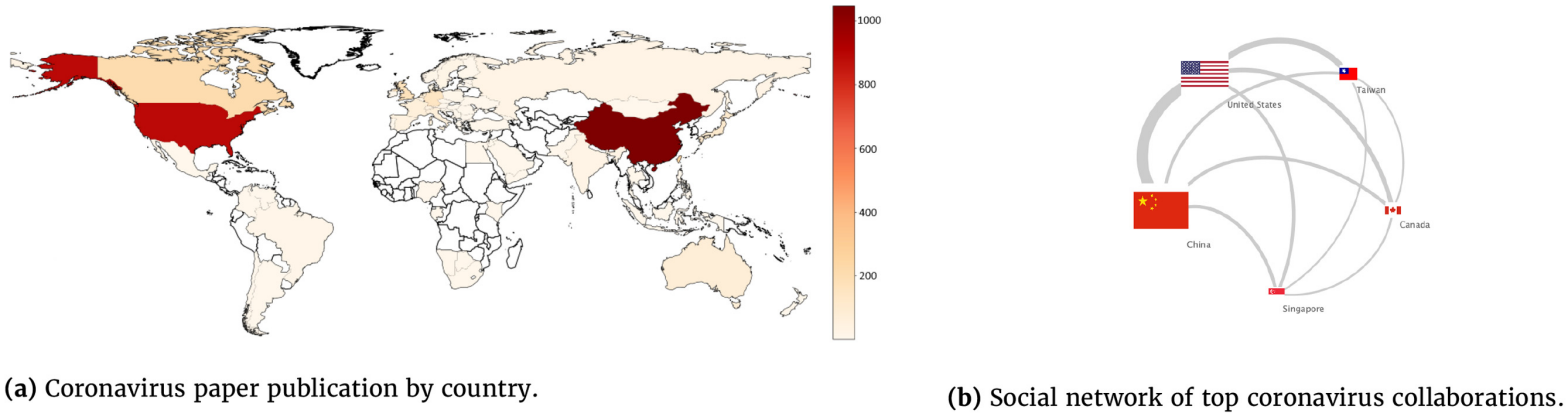
International research on the coronavirus.

By inspecting global collaboration and research efforts, we found that the geolocation of researchers correlated with publication trends. For instance, most SARS, MERS, hepatitis B, and avian influenza research was done by investigators based in the USA and China (Fig. 7). In the case of SARS and MERS, most of the research stemmed from China and the USA (Fig. 8), with only 17% of SARS papers' first authors being located in Europe. Overall, researchers from 57 and 67 countries have studied MERS and SARS, respectively. However, most SARS papers (73%) were written by researchers in only 6 countries (Fig. 7). While the USA was dominant in the research of all inspected diseases, China showed an increased output in only these 3 diseases. Also, MERS and SARS were studied in the fewest countries, and HIV was studied in the most countries (Fig. 7). Moreover, SARS and MERS were the diseases least studied in Europe, with only 17% and 19% of SARS and MERS studies, respectively, as opposed to Ebola studies, 29% of which were conducted in Europe.

**Figure 9:**
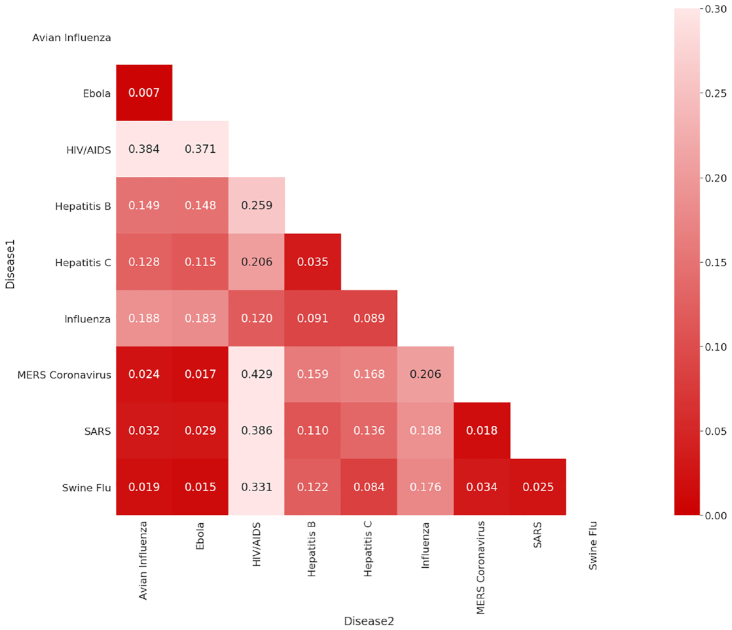
DTW distance between NPR of diseases.

**Figure 10:**
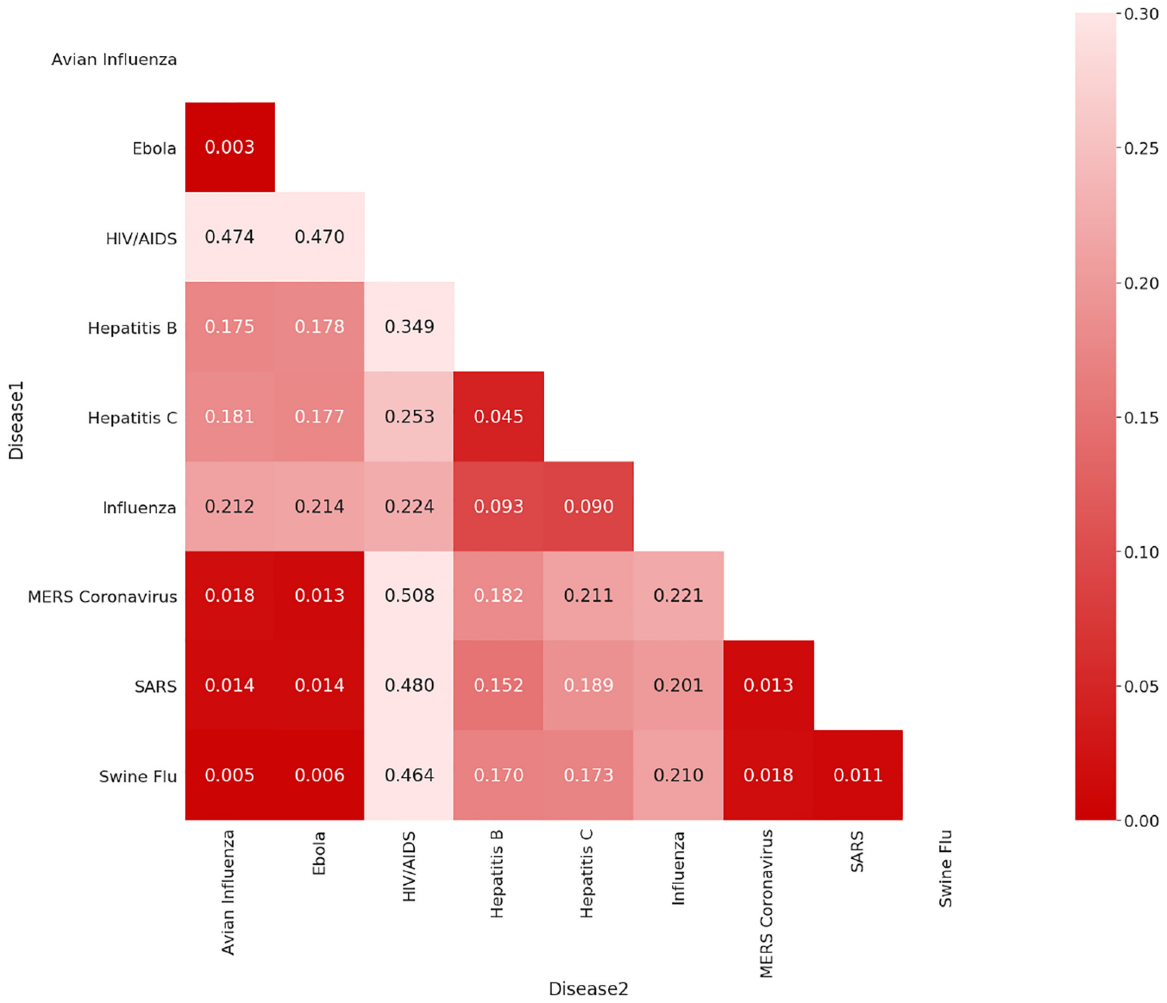
DTW distance between NCR of diseases.

## Discussion

In this study, we analyzed trends in the research of emerging viruses over the past 2 decades with emphasis on emerging coronaviruses (SARS and MERS). We compared the research of these 2 coronavirus epidemics to 7 other emerging viral infectious diseases as comparators. To this end, we used multiple bibliometric datasets, fusing them to get additional insights. Using these data, we explored the research of epidemiology from the perspectives of papers, journals, authors, and international collaborations.

By analyzing the results presented in the Results section, the following can be noted: First, the surge in infectious disease publications (Fig. 1) supports the results of Fire and Guestrin [36], who found that there has been a general escalation of scientific publications. We found that the increase in the number of infectious disease publications is very similar to that in other fields. Hence, Goodhart's Law (“When a measure becomes a target, it ceases to be a good measure”) did not skip the world of virology research. However, alongside the general increase in the number of papers, we observed that there was a decline in the relative number of papers on the specific infectious diseases that we inspected. The most evident drastic decrease in the publication rate happened after an epidemic ended. It seems that, for a short while, many researchers study an outbreak, but later their efforts are reduced. This finding is strengthened by considering the average number of multiple papers per author for each disease (see Table 2). Additionally, similar patterns were found in the funding of MERS and SARS research [25], which indicates that there is a possibility that the research rate has decreased as a result of lack of funding.

**Table 2:** Mean papers published by author with multiple papers related to a specific disease

Disease	Papers
Swine flu	3.45
SARS	3.84
MERS Coronavirus	3.86
Ebola	4.07
Hepatitis B	4.42
Avian influenza	4.47
Influenza	5.04
Hepatitis C	5.24
HIV/AIDS	6.31

Second, when looking at journal publications, we noted that very similar patterns occurred for citations and publications. This result emphasizes that fewer publications, and hence fewer citations, translate into fewer papers in Q1 journals (Fig. 4). Also, we observed the same patterns as Fire and Guestrin [36], with most of the papers being published in Q1 journals and the minority published in Q2–Q4 journals. This trend started to change when zooming in and analyzing publications in top-10–ranked journals (Fig. 5). While we can see some correlation to outbreaks in Ebola, swine flu, and SARS, it is harder to interpret the curve of HIV because there were no focused epidemics in the past 20 years but a global burden, and we did not observe similar patterns in publications and citations. Observing the JScore (Journal Trends section) results (Fig. 6), most diseases showed a steady increase, but 2 diseases behaved rather anomalously. MERS had a decline since 2013, which is reasonable to expect after the initial outbreak, but we did not see the same trend in the other diseases and there is a general trend of increasing average SJR [36]. The second anomaly is that SARS had an increase in JScore alongside a decrease in citations and publication numbers. Inspecting the data, we discovered that in 2017 there were 3 published papers in *Lancet Infectious Diseases* and in 2015 2 papers in *Journal of Experimental Medicine* about SARS, and both journals have a very high SJR. These publications increased the JScore drastically. This anomaly is a result of outliers in the data that biased the results. We can observe in Fig. 4 that in the past decade the number of SARS papers published in ranked journals decreased drastically. It dropped low enough that 2 outliers created a bias on the JScore. Generally, the less data we have, the greater chance for outliers to cause bias in the data.

Third, we observed that on average authors write fewer multiple papers on diseases that are characterized by large epidemics, such as the swine flu and SARS. On the other side of the scale are hepatitis C and HIV, which are persistent viral diseases with high global burdens. These diseases involve more prolific authors. Regarding Ebola and MERS, it is too early to predict whether they will behave similarly to SARS because they are relatively new and require further follow-up.

Fourth, looking at international collaboration, we observed the USA to be very dominant in all the disease studies (Fig. 7). Looking at China, we found it to be mainly dominant in diseases that were epidemiologically relevant to public health in China, such as SARS, avian influenza, and hepatitis B. When looking at Ebola, which has not been a threat to China for the past 2 decades, we observed a relatively low investment in its research in China. We observed that regarding MERS, we found results similar to those of Sa'ed [50]. In both studies the top 3 biggest contributors in MERS studies were the USA, China, and Saudi Arabia.

Many of the trends that we observed are related to the pattern of the diseases. We observed 2 main types of infectious diseases with distinct trends. The first type was emerging viral infections such as SARS and Ebola. Their academic outputs tend to peak after an epidemic and then subside. The second type were viral infections with high burdens such as hepatitis B and HIV, for which there is a more or less constant trend. These trends were most evident in publication and citation numbers, as well as journal metrics. The collaboration and author distributions were more affected by where the outbreak occurred or where there was a high burden. This was also strengthened in the clusters we found, where they were divided in the same way.

In terms of practical implications, we see several options. First, notwithstanding the importance of pathogen discovery, as evident in projects like the Global Virome Project [51] that is trying to discover unknown zoonotic viruses to stop future outbreaks, it is still important to monitor the status of current research that concerns known pathogens. It can be observed from Figs 2 and 3 that there are diseases with declining interest from the scientific community. These trends are harder to spot when looking at the total number of publications because the total number of papers generally keeps increasing (Fig. 1a). Using NPR and NCR can help decision makers investigate whether additional resources should be invested in the study of these diseases. For instance, while SARS and MERS were in WHO's R&D Blueprint as priority diseases, they still exhibited a decline in their research rate. Second, using collaboration data, it is possible to find which countries have potential for growth in the number of researchers on specific diseases and also which bilateral grants have potential.

Currently, there is no doubt that we have to be better prepared for the next pandemic and the emergence of “Disease X.” We observed that currently there is a non-sustained investment in EIDs such as SARS and MERS, which is a key issue. Another crucial issue is the sharing of research material such as data and code. Data and code allow scientists to make more accurate discoveries faster by continuing knowledge from previous studies. Using the MAG dataset Paper Resources table, we inspected how many papers from the 9 diseases we analyzed had code or data. We found that there were 30 and 75 papers that had data and code, respectively. These numbers are very low, and we suspect that there are a lot of missing data in this table. We firmly believe that publishing code and data should be mandatory when possible.

**Figure 11:**
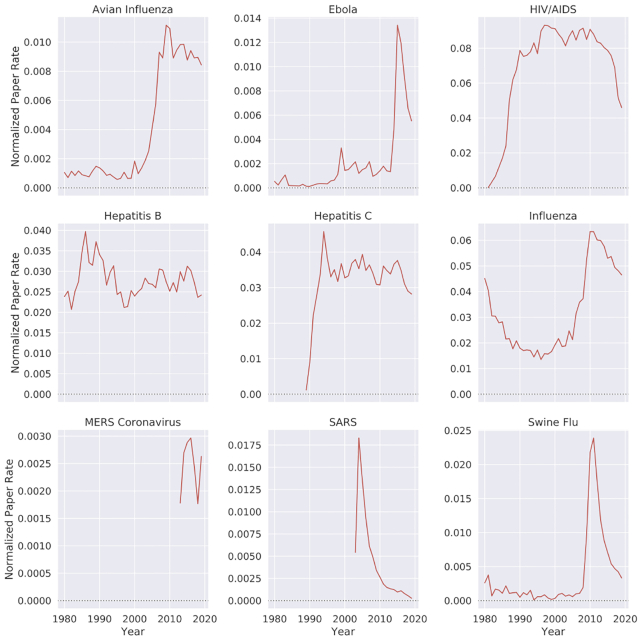
Normalized paper rate of the virology category by different diseases over time.

**Figure 12:**
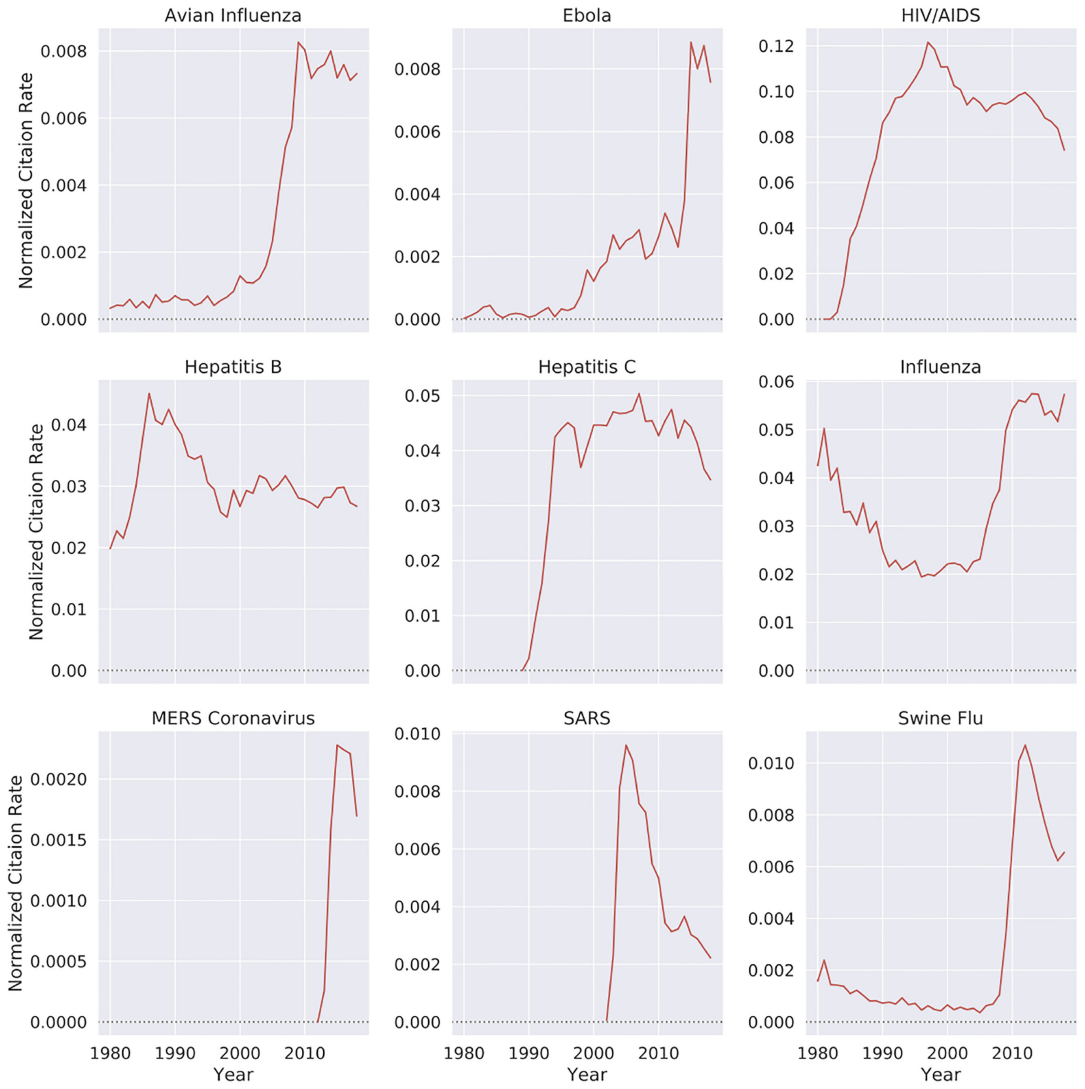
Normalized citation rate of the virology category by different diseases over time.

This study may have several limitations. To analyze the data, we relied on titles to associate papers with diseases. While a title is very important in classifying the topic of a paper, some papers may discuss a disease without mentioning its name in the title. Additionally, there may be false-positive hits; for instance, an acronym might have several meanings that are not related to an infectious disease term. An additional limitation is our focus on a limited number of distinct diseases. There are other emerging infections not evaluated herein that could have followed other trends. To deal with some of these limitations, we only analyzed papers that were categorized as medicine and biology papers as a means to reduce false-positive results. Furthermore, we show that the same trends appeared even when we filtered all the papers by the category of virology (see Figs 11 and 12). Finally, we compared papers that were tagged with a MeSH term on PubMed to the papers we retrieved using our keyword search of the title. We found that we matched MeSH terms with 73% recall, which is in the range described by Breugelmans et al. [24].

In the future, we would like to perform extended collaboration analysis by improving the institution country mapping. Currently, we were able to identify 94% of the countries of origin for the institutions in the MAG affiliation table. We intend to improve the institution country mapping by using additional data sources. In addition, we are planning to extend our study into other diseases and look for correlations with real-world data such as global disease burden.

## Conclusions

The COVID-19 outbreak has emphasized the insufficient knowledge available on emerging coronaviruses. Here, we explored how previous coronavirus outbreaks and other emerging viral epidemics have been studied over the past 2 decades. From inspecting the research outputs in this field from several different angles, we demonstrate that the interest of the research community in an emerging infection is temporarily associated with the dynamics of the incident and that a drastic decrease in interest is evident after the initial epidemic subsides. This translates into limited collaborations and a non-sustained investment in research on coronaviruses. Such a short-lived investment also involves reduced funding as presented by Head et al. [25] and may slow down important developments such as new drugs, vaccines, or preventive strategies. There has been an unprecedented explosion of publications on COVID-19 since January 2020 and also a significant allocation of research funding. We believe the lessons learned from the scientometrics of previous epidemics argue that regardless of the outcome of the COVID-19 pandemic, efforts to sustain research in this field should be made. More specifically, in 2017 [52] and 2018 [53], SARS and MERS were considered to be priority diseases in WHO's R&D Blueprint, but their research rate did not grow relative to other diseases. Therefore, the translation of international policy and public health priorities into a research agenda should be continuously monitored and enhanced.

## Availability of Source Code and Requirements

Project name: ScienceDynamics

Project home page: https://github.com/data4goodlab/ScienceDynamics

Operating system(s): Linux, OS X

Programming language: Python

Other requirements: Python 3.6 or higher

License: MIT License

RRID:SCR_018819

## Availability of Supporting Data and Materials

Supporting data and a copy of the code are available via the *GigaScience* GigaDB Database [54].

## Abbreviations

COVID-19: novel coronavirus disease 2019; DTW: dynamic time warping; EID: emerging infectious disease; JCR: Journal Citation Reports; MAG: Microsoft Academic Graph; MERS: Middle East respiratory syndrome; MeSH: Medical Subject Heading; NCR: Normalized Citation Rate; NPR: Normalized Paper Rate; SARS: severe acute respiratory syndrome; SARS-CoV-2: severe acute respiratory syndrome coronavirus 2; WHO: World Health Organization.

## Competing Interests

The authors declare that they have no competing interests.

## Authors' Contributions

All the authors conceived the concept of this study and developed the methodology and contributed to its writing. D.K. developed the study's code and visualization and performed the data computational analysis. J.M.G reviewed and edited the manuscript. M.F. supervised the research.

## Supplementary Material

giaa085_Supplemental_Files

giaa085_GIGA-D-20-00084_Original_Submission

giaa085_GIGA-D-20-00084_Revision_1

giaa085_GIGA-D-20-00084_Revision_2

giaa085_GIGA-D-20-00084_Revision_3

giaa085_Response_to_Reviewer_Comments_Original_Submission

giaa085_Response_to_Reviewer_Comments_Revision_1

giaa085_Response_to_Reviewer_Comments_Revision_2

giaa085_Reviewer_1_Report_Original_Submission Nicola Luigi Bragazzi -- 3/24/2020 Reviewed

giaa085_Reviewer_2_Report_Original_Submission Daniel Mietchen -- 4/15/2020 Reviewed

giaa085_Reviewer_2_Report_Revision_1 Daniel Mietchen -- 6/26/2020 Reviewed

giaa085_Mietchen_Review1_GIGASCIENCE_GIGA_D_20_00084_suppl

giaa085_Mietchen_Review2_GIGASCIENCE_GIGA_D_20_00084R1

giaa085_Review_Response

giaa085_Review_Response2
