# Supplementary material for: Scientometric trends for coronaviruses and other emerging viral infections
Source: Gigascience. 2020 Aug 17;9(8):giaa085. doi: 10.1093/gigascience/giaa085 (PMC7429184; doi:10.1093/gigascience/giaa085)
Supplement: giaa085_Mietchen_Review1_GIGASCIENCE_GIGA_D_20_00084_suppl [file giaa085_mietchen_review1_gigascience_giga_d_20_00084_suppl.zip › Virology_NCR.pdf]

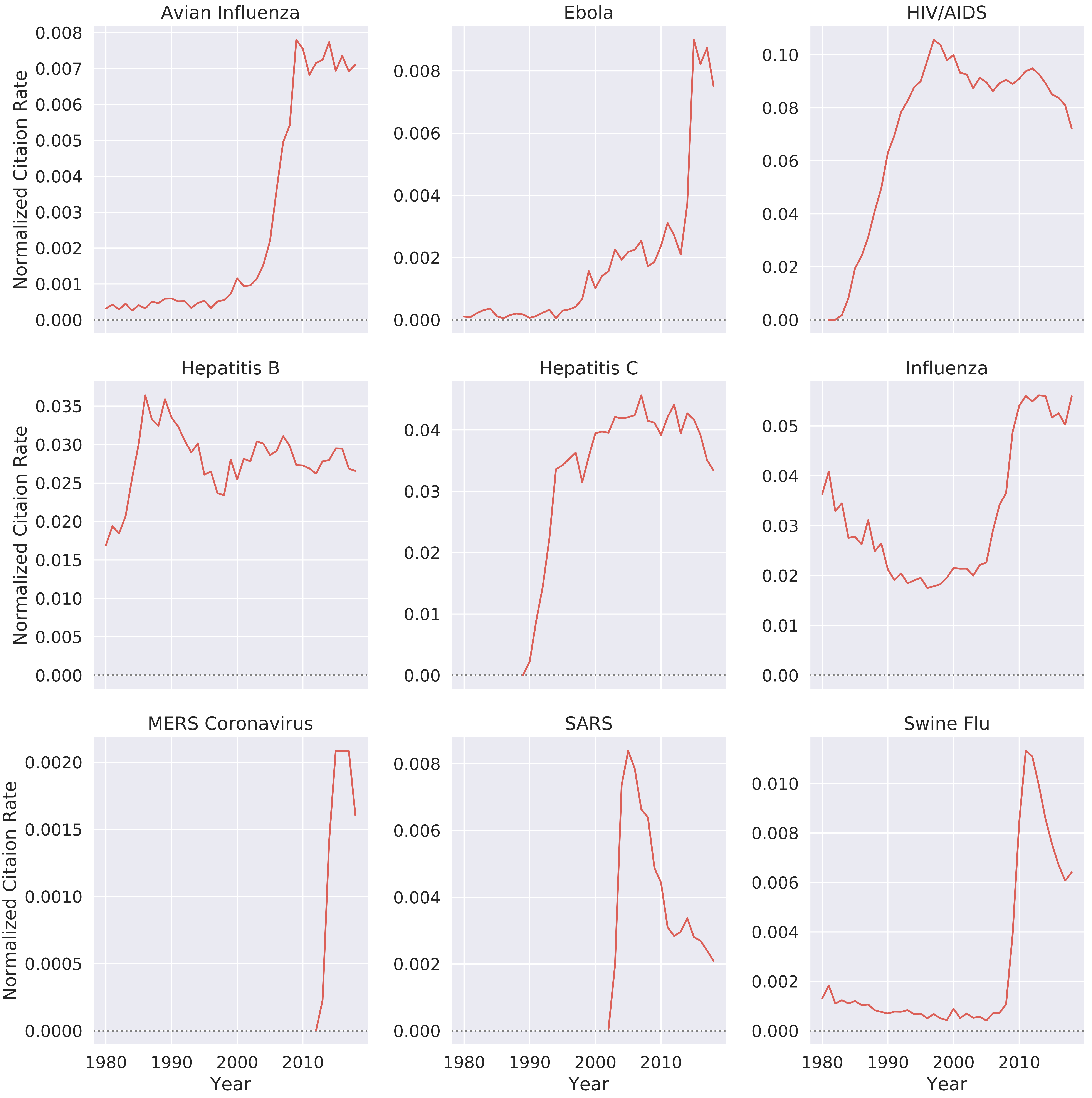

Avian Influenza
Ebola
HIV/AIDS
Hepatitis B
Hepatitis C
Influenza
MERS Coronavirus
SARS
Swine Flu
Normalized Citaion Rate
Year
1980
1990
2000
2010
