# Supplementary figures and images for: Scientometric trends for coronaviruses and other emerging viral infections
Source: Gigascience. 2020 Aug 17;9(8):giaa085. doi: 10.1093/gigascience/giaa085 (PMC7429184; doi:10.1093/gigascience/giaa085)

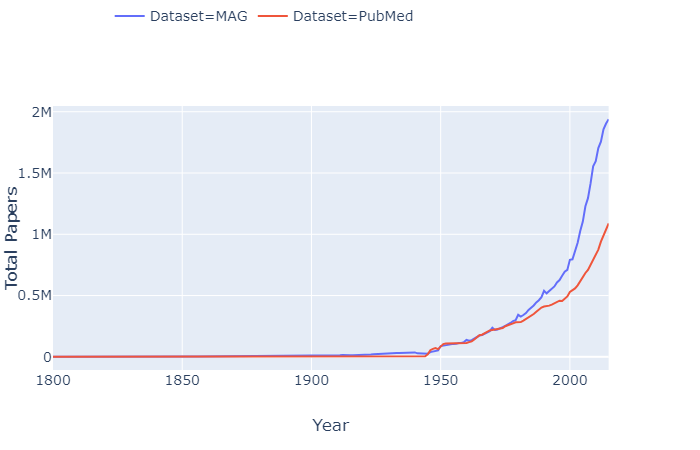

Supplement: giaa085_Mietchen_Review1_GIGASCIENCE_GIGA_D_20_00084_suppl [file giaa085_mietchen_review1_gigascience_giga_d_20_00084_suppl.zip › Total Papers.png]

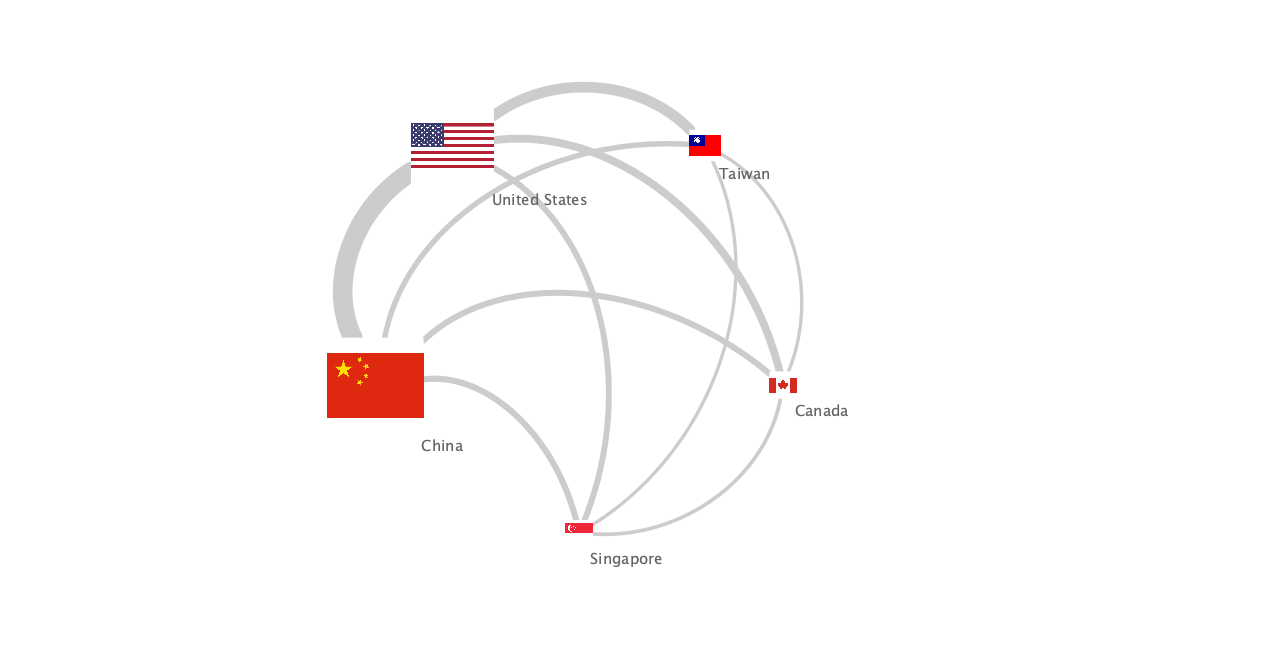

Supplement: giaa085_Mietchen_Review1_GIGASCIENCE_GIGA_D_20_00084_suppl [file giaa085_mietchen_review1_gigascience_giga_d_20_00084_suppl.zip › SARS-net.png]

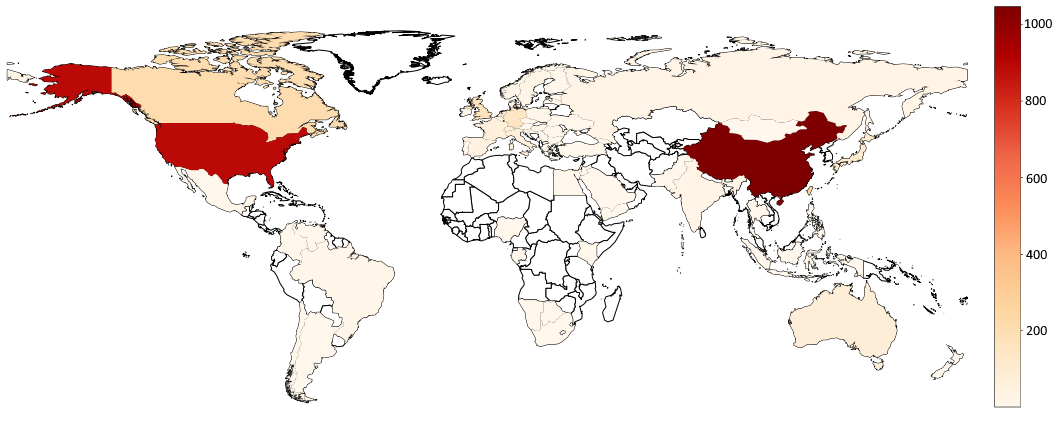

Supplement: giaa085_Mietchen_Review1_GIGASCIENCE_GIGA_D_20_00084_suppl [file giaa085_mietchen_review1_gigascience_giga_d_20_00084_suppl.zip › map.png]

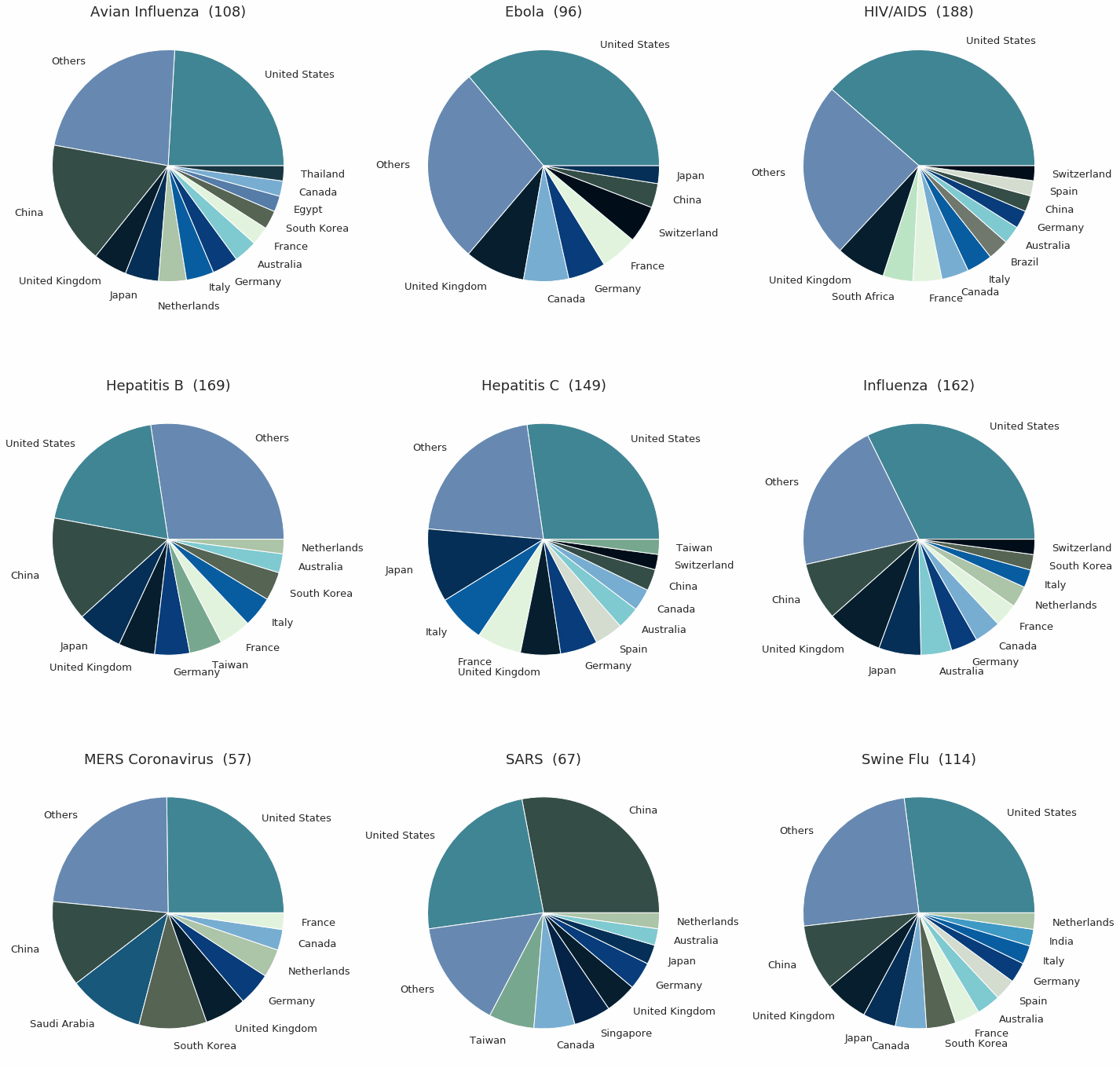

Supplement: giaa085_Mietchen_Review1_GIGASCIENCE_GIGA_D_20_00084_suppl [file giaa085_mietchen_review1_gigascience_giga_d_20_00084_suppl.zip › inter.png]

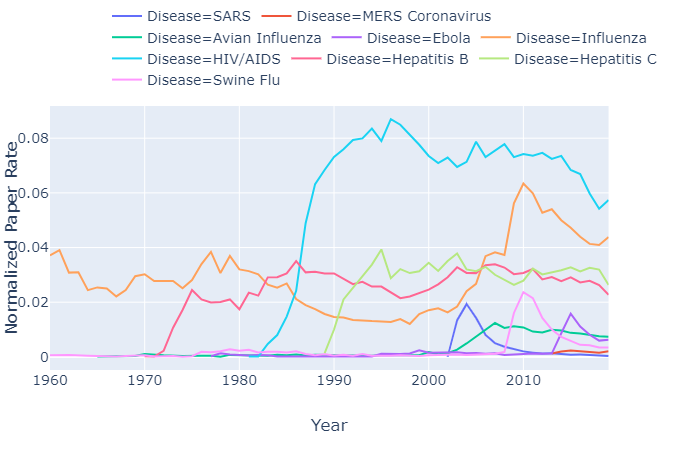

Supplement: giaa085_Mietchen_Review1_GIGASCIENCE_GIGA_D_20_00084_suppl [file giaa085_mietchen_review1_gigascience_giga_d_20_00084_suppl.zip › disease.png]
