# Supplementary material for: Scientometric trends for coronaviruses and other emerging viral infections
Source: Gigascience. 2020 Aug 17;9(8):giaa085. doi: 10.1093/gigascience/giaa085 (PMC7429184; doi:10.1093/gigascience/giaa085)
Supplement: giaa085_Response_to_Reviewer_Comments_Original_Submission [file giaa085_response_to_reviewer_comments_original_submission.pdf]

Author's Response To Reviewer Comments

Close

Please see the attached Review-Response.pdf file.

Close
