# Supplementary material for: Scientometric trends for coronaviruses and other emerging viral infections
Source: Gigascience. 2020 Aug 17;9(8):giaa085. doi: 10.1093/gigascience/giaa085 (PMC7429184; doi:10.1093/gigascience/giaa085)
Supplement: giaa085_Response_to_Reviewer_Comments_Revision_1 [file giaa085_response_to_reviewer_comments_revision_1.pdf]

Author's Response To Reviewer Comments

Close

Please see attached file: Review-Response2.pdf

Close
