# Supplementary material for: Scientometric trends for coronaviruses and other emerging viral infections
Source: Gigascience. 2020 Aug 17;9(8):giaa085. doi: 10.1093/gigascience/giaa085 (PMC7429184; doi:10.1093/gigascience/giaa085)
Supplement: giaa085_Response_to_Reviewer_Comments_Revision_2 [file giaa085_response_to_reviewer_comments_revision_2.pdf]

Author's Response To Reviewer Comments

Close

1. main thing is to cite the data DOI in the data section of the paper - Added citation to GigaDB.

2. In addition, please include the RRID of the tool in your manuscript. This will facilitate tracking, reproducibility and re-use of your tool. - Added RRID in the Availability of source code and requirements section

3. Can you add ORCID details for any authors that have them? - ORCID was added for all authors. We tried to add it as an icon but we got exceptions that are related to the \author command.

4. Delete these three words - deleted.

5. We want URLs cited in the references - All URLs in the footnotes were converted into citations.

Close
