# Supplementary material for: Scientometric trends for coronaviruses and other emerging viral infections
Source: Gigascience. 2020 Aug 17;9(8):giaa085. doi: 10.1093/gigascience/giaa085 (PMC7429184; doi:10.1093/gigascience/giaa085)
Supplement: giaa085_Review_Response [file giaa085_review_response.pdf]

"Scientometric Trends for Coronaviruses and Other Emerging Viral Infections"- Response to Reviewers

We would like to thank the reviewers for their highly valuable and constructive criticism. The comments have been very helpful in the preparation of the revised manuscript. We have addressed the reviewers' concerns and have improved the paper accordingly. Moreover, we invested considerable efforts to make our study and code easy to reproduce. Furthermore, we generated a ready to run docker image [1]. The following is a description of the revisions we have made to address the comments pointed out by the reviewers. Please note that we have also provided the revised paper in which we have marked all changes in blue.

Reviewer #1:

Comment 1:
Figures are of low quality and I have difficulties in properly seeing them; can you make them of higher resolution and more readable;

Response 1:
We thank the reviewer for pointing this out. In the revised manuscript we have improved the figures: First, the figures were re-generated with higher resolution. Second, we adjusted the fonts to improve the readability of several figures.

Comment 2:
There is a part dedicated to the so-called non-conventional data streams, like google trend, wiki, etc., I would shorten this part, since it is a little bit out of the scope and focus of the paper,

Response 2:
We have shortened the referenced parts according to the reviewer's suggestion.

Comment 3:
Besides figures, can authors provide more quantitative data and statistical analyses (of signficance) if possible

Response 3:
Comparing temporal graphs is a challenging task. We tried to use regression algorithms to model the curves. However, we did not obtain any significant insights using this method. Different diseases behave differently; for instance, a signal of Disease A can be slower than Disease B since it spreads faster or slower, but in general, they can have the same pattern. Due to this reason, to compare the plots we used DTW (dynamic time warping), a signal processing method to measure the similarity between temporal sequences that vary in speed. Additionally, we used TimeSeriesKMeans, a method based on KMeans clustering that utilizes DTW to measure the distance to cluster the plots/time series into groups. We discovered that there are two clusters: the first contains avian influenza, Ebola, MERS, SARS, and

swine flu, and the second cluster contains HIV/AIDS, hepatitis B, hepatitis C, and influenza.

Comment 4:
Can authors put their research in a broader view and also in terms of practical implications (for example, the more research, the higher the likelihood of finding a cure, etc.). can authors correlate bibliometric trends with outcomes

Response 4:
We see here several practical implications. First, we can use the research to find diseases that are understudied. For instance, SARS and MERS were considered in WHO's R&D blueprint as priority diseases. While looking in PubMed, it is easy to conclude that the current amount of SARS research is steady. However, since there is a constant growth in the number of published papers in almost all topics, there is an overall decrease in the percentage of papers about SARS. In other words, even with SARS being considered as a priority disease, there is a decline in the publication rate of SARS papers. Parallel to the drop in the number of papers, there has also been a drop in funding [2] of this coronavirus. After the beginning of the outbreak, reports came in about a scientist that looked for a coronavirus vaccination years ago but stopped the research due to lack of funding and interest from the scientific community [3]. Hence, a drop in the number of papers can indicate reduced interest from the scientific community or diminished resource allocation. We believe further research is needed on the topic to be able to prove causality. Second, using the geodata, we can see which countries have the potential to attract new scientists to study specific diseases. To address these topics, we have added material in our Discussion section.

Reviewer #2:

Comment 1: (page 1)
Please ensure that all cited URLs are properly archived, preferably via http://web.archive.org/save/

Response 1:
As the reviewer suggested, we have made sure that all the URLs were saved via http://web.archive.org/save/.

Comment 2: (page 1)
not using DOIs in references when they are available makes scientometric analyses like this one more error-prone

Response 2:
We have now added DOIs to all the referenced academic papers.

Comment 3: (page 1)
That's the name of the disease, not the virus

Response 3:
Thank you for pointing this out; in the new revision we have resolved this issue.

Comment 4: (page 1)
Consider updating these numbers during revision

Response 4:
As suggested, we have updated the numbers.

Comment 5: (page 2)
I'm not convinced having such a results part in the introduction is useful and could not find guidance on that in the Instructions to Authors. Perhaps expand the results part in the abstract a bit, but otherwise, this paragraph (and basically all of the remainder of the introduction) probably belongs into the results section (where it essentially already is).

Response 5:
Thank you for pointing this out; in the revised paper we have merged this paragraph with the relevant Results subsections.

Comment 6: (page 2)
Are two significant digits appropriate here?
Response 6:
We have updated the numbers to five digits.

Comment 7: (page 2)
perhaps noteworthy in this context that there were some cases in 2004 as well, e.g. as per SARS in China

Response 7:
The reviewer is correct. Even though SARS was considered contained in July 2003, there was a small outbreak in 2004 (nine cases). In the revised manuscript, we have updated the years to be 2002-2004, according to the reviewer's suggestion.

Comment 8: (page 2)
It's odd to see Figure 8 mentioned before Figure 2

Response 8:
This paragraph was transferred into the Results section, solving this issue.

Comment 9: (page 2)
Consider including internal links to facilitate navigation.

Response 9:
As the reviewer suggested, we have included internal links in all the missing places.

Comment 10: (page 2)
Not sure what this sentence adds. If you think the point is important, a bit more detail would be needed.

Response 10:
Thank you for noticing this; we have removed the sentence.

Comment 11: (page 3)
Similar studies have been performed using Wikipedia pageview stats. Such data share many of the problems discussed above but are open by default and available in standard formats for well over a decade into the past.

Response 11:
Thank you for pointing this out. We have expanded the Infectious Disease Trends subsection to also reference Wikipedia-based studies.

Comment 12: (page 5)
Would be useful to have some explanation somewhere about whether and how author disambiguation was performed.

Response 12:
We have added an explanation to note that the authors were identified by the identification number provided in the MAG dataset. Microsoft combined entity matching with data extracted from academics' CVs to generate their author IDs [4]. Additionally, they allow authors to claim their papers and to fix errors in the algorithm.

Comment 13: (page 6)
Including image files as PDF complicates the generation of a useful HTML later on.

Response 13:
As suggested, we have converted all the image files to high-resolution PNG files.

Comment 14: (page 6)
ref{fig:papers1} does not provide any information about infectious diseases, so the integration of text and figure can be improved

Response 14:
We thank the reviewer for this comment. In the revised manuscript, the integration text was improved accordingly.

Comment 15: (page 6)
somewhere, perhaps here, a comment on research on other coronaviruses would make sense

Response 15:

Indeed, there are other strains of coronaviruses that cause seasonal respiratory infections. Since these strains do not cause outbreaks, we focused only on SARS and MERS. We have added a comment regarding research on other coronaviruses in the Analyses section.

Comment 16: (page 7)
That is widely discussed in the literature, so adding some pointers here would be useful.

Response 16:
We agree with the reviewer that there are many papers about international collaboration. However, we did not find studies that focused on measuring worldwide infectious disease collaborations. We only found papers that discussed the topic in general, or in a specific field. In the case of the relevant infectious diseases, we found one paper that analyzed MERS publications. We have added to the Discussion section the similarity we observed about MERS research.

Comment 17: (page 7)
China`s share in Hepatitis B papers is higher than its share in MERS papers.

Response 17:
Thank you for bringing this to our attention, and we have revised the text accordingly.

Comment 18: (page 8)
Good occasion to discuss the role of biases and outliers in the data more generally

Response 18:
We agree with the reviewer's comment, that it is a good idea to discuss the outlier, and we have now addressed this in the Discussion section as the reviewer suggested.

Comment 19: (page 8)
Assuming the acronym stands for an infectious disease, wouldn`t the failure to recognize that be a false negative?

Response 19:
The original intention was that acronym may have several meanings, and there is a possibility of misclassifying papers as infectious disease related. The sentence was rephrased to make this clearer.

Comment 20: (page 9)
The Global Virome Project aims to address viruses systematically.

Response 20:
As the reviewer suggested, we have now mentioned this important project.

Comment 21 (page 5)
In any case, it would be useful to state from which Wikipedia you extracted this information. I assume it was the English one. Chances are, though, that for

institutions outside the English-speaking world, a non-English Wikipedia might have better location information than the English one.

Response 21
We indeed used the English Wikipedia. In the revised manuscript, we have added a footnote mentioning that we utilized the English Wikipedia. The idea of using a non-English Wikipedia to get information that is missing in the English Wikipedia is very interesting, and we intend to check it out in future studies. Using only the English Wikipedia and Wikidata, we were able to find the countries of 94.2% of the institutions in the MAG affiliation table. We have added to the Discussion section information about the accuracy of our mapping and that we are planning to improve it in future studies.

Comment 22 (page 5)
What is the underlying dataset~-- MAG or PubMed or both? This should be indicated. In any case, the individual graphs are also available from Pubmed, e.g. Ebola, so you could compare against them. Not sure the repetitive ``Disease='' marker is helpful in the legend; that seems more appropropriate for the caption.}

Response 22
Figure 1b contains data from the MAG dataset, and we have updated the figure and the caption according to the reviewer's suggestion. Regarding the graphs available on PubMed, they present the total number of papers while our graphs show the normalized amount.

Comment 23: (page 8)
Why not take MeSH terms?

Response 23:
During our research, we considered using MeSH terms and even at some point PubMed was our primary dataset. However, we discovered that PubMed lacks many fields, for instance, paper citations. Additionally, other fields such as author's affiliation are very noisy. We tried to deal with these problems, but the results were inadequate. Generally, the MAG dataset is larger and has more information than PubMed (Figure 1a); for instance, it has citation, affiliation, author disambiguation, etc., while all these data are missing in PubMed. For all the analyses except the journal trends, MAG presented a better option than PubMed, and the only shortcoming was that the MAG dataset did not have MeSH terms. To present a unified method in the paper, we used the same method for MAG and the journal trends analysis where we used PubMed. Moreover, we compared paper retrieval by title and MeSH, and 73% of the retrieved papers were the same. A similar problem with the MeSH terms is described by Breugelmans et al. [5]. They also arrived at similar recall values between MeSH terms and keyword search on an external dataset.

Comment 24: (page 8)
yes, but a quick PubMed search like https://pubmed.ncbi.nlm.nih.gov/?term=zika can be used to check that, and the one at https://pubmed.ncbi.nlm.nih.gov/?term=coronavirus in a way sums up the paper.

Response 24:
We understand the reviewer's concerns. However, using the PubMed search to check trends in diseases is problematic. There are more than 300 infectious diseases with many aliases; manually inspecting all these diseases is not efficient and will not provide a complete graph. The same problem exists even when performing search using MeSH terms; Breugelmans et al. [5] mapped multiple MeSH terms for each disease in order to search for papers. Moreover, the data presented on PubMed is not normalized, and since the total number of published papers is growing every year in the long run, we will see on PubMed a trend of growth almost for every topic.

Comment 25: (page 8)
some reference(s) on the funding would be useful here, e.g. some of those at http://researchinvestments.org/publications. Their pneumonia report at http://researchinvestments.org/pneumonia/ is rather detailed but does not mention coronavirus, which helps make the case for your paper. They do have a supplementary database, though, that has some coronavirus-related information, as per https://aqueous-inlet-57355.herokuapp.com/?disease_areas__name=Coronavirus\&breakdown=type_of_research

Response 25:
We agree with the reviewer that funding data may strengthen the paper results. While the paper was under review, a new pre-print was published [2] that analyzed infectious disease funding. This paper's results present similar patterns to the patterns we discovered in paper publications and citations. We have added this information to the paper as the reviewer suggested.

Comment 26: (page 9)
good place to refer to the WHO's R&D Blueprint

Response 26:
As the reviewer suggested, we have added a reference to the WHO's R&D Blueprint. Surprisingly, we discovered that according to the Blueprint, SARS and MERS were considered to be priority diseases in 2017 and 2018 but that did not increase the research rate.

Comment 27: (page 9)
The paper focuses on research on (some) human coronaviruses, without mentioning others. So perhaps adapt this question a bit, or add a section with some pointers to research on other coronaviruses, perhaps separately for human/ lifestock/ wild animals.

Response 27:
Thank you for pointing this out. We have changed the question accordingly.

Comment 28: (page 9)
the paper zooms in on a few selected cases from which it is hard to generalize to the level of this question, so the paper only addresses some aspects of it. It also does not consider trends in publications on EIDs as an abstract, cross-disease concept (think ``Disease X'' or ``next pandemic'')

Response 28:
The reviewer is correct in that we focused on selected diseases and our findings are not generalizable to all infectious diseases. Our paper shows a proof of concept on how scientometric analyses could support such investigations and definitely leaves room for future analyses focusing on other diseases. As for EIDs as a cross-cutting concept, we agree this merit further research, but in our opinion other methods should be employed to answer such a question (e.g., NLP) and it is beyond the scope of the current analysis.

Comment 29: (page 9)
the applied part here is thin. One application I see that is not discussed is a greater recognition of the need for sharing data and code as well as preprints, which means, for instance, that scientometric studies that want to capture publications during the pandemic will need to consider whether and how to include such materials.

Response 29: (page 9)
The biggest application we see here is that the detection of understudied diseases can encourage the allocation of additional funds for these diseases to increase research volume as appropriate. We also believe that the sharing of code and data is very important for driving science forward. We used the MAG dataset to inspect how many papers include code and data. However, the numbers we found were very low, 30 and 75 papers had data and code, respectively. We did not include this since we suspect that this a result of incomplete data in the relevant MAG table. In the revised paper, we have added two paragraphs on this issue in the Discussion section.

Comment 30: (page 9)
Similarly, Wikidata could assist with much more of this work than just the geolocation of the institutions. For an overview, see Wikidata as a knowledge graph for the life sciences. As for concrete examples, this Scholia profile provides a Wikidata-based overview of the COVID-19 literature, and this demo illustrates a Wikidata way of creating your Fig.\ \ref{fig:net}. The Scholia profile also has an accompanying page detailing some of the known gaps associated with it, so as to facilitate collaborative curation. It would be very helpful to be able to feed the curation work that you have already undertaken on this corpus into this shared system.

Response 30:

This is the first time we have stumbled upon Scholia. The project looks very interesting with a lot of potential to move forward bibliometric studies. We advocate for open source, we would gladly feed the data we have to Scholia/Wikidata; for instance, we noticed that some geolocation data can be quite easily improved. We assume that geolocation data can be modified by a bot on Wikidata. We would gladly incorporate our data with the project.

Comment 31: (page 9)

While looking beyond classical publications and since you are using Wikipedia already, it may be worth considering its role as a health information resource, the impact of news exposure on collective attention during an epidemic and patterns of reader engagement with medical content on Wikipedia, especially in the current pandemic and in light of the accompanying misinformation problems

Response 31:
As the reviewer suggested, we have noted in the Background section the role of Wikipedia as a medical data source in general, as well as in the current outbreak.

Comment 32: (page 9)
I could not access the private GitHub repo, as it was set to dual authentication, of which I only had one part. For the version of the code that I got from the FTP server, I could not get it to run under any of the systems I tried (a Linux laptop, a MacBook and under \href{https://www.mediawiki.org/wiki/PAWS}{PAWS}), due to issues with the dependencies. Providing the code in a containerized form (e.g. via \href{https://github.com/minrk/ligo-binder}{Binder}) would be very useful in terms of reproducibility and speeding up the review. In addition to that, a copy of the code should be archived (e.g.\ with Zenodo) and cited from the paper.

Response 32:
Thank you for trying to run our code; it is very important to us that our studies be reproducible. The code has now been refactored and updated to be easier to run and understand. Additionally, we updated the installation instructions (please see the README file). As the reviewer suggested, we will try using binder when we make the project public. To make the code easier to execute we created a docker image with all the requirements installed. The docker image and all the minimal required data (to reduce the upload time) was uploaded to a public google drive folder [1]. If the paper is accepted, we will upload all the code and raw data to the GigaScience repository.

Bibliography

[1] [Online]. Available: https://drive.google.com/drive/folders/1XYbRY9g_9qjfA45j0pAFnX1oCWhQPWfY?usp=sharing.

[2] M. G. Head, R. J. Brown,, M.-L. Newell, A. J. Scott, J. Batchelor and R. Atun, "The Allocation of US$ 105 Billion in Global Funding for Infectious Disease Research between 2000 and 2017: An Analysis of Investments from Funders in the G20 Countries," 2020.

[3] "Scientists were close to a coronavirus vaccine years ago. Then the money dried up.," NBC News, 2020. [Online]. Available: https://www.nbcnews.com/health/health-care/scientists-were-close-coronavirus-vaccine-years-ago-then-money-dried-n1150091.

[4] "https://www.microsoft.com/en-us/research/project/academic/articles/microsoft-academic-uses-knowledge-address-problem-conflation-disambiguation/," [Online]. Available: https://www.microsoft.com/en-us/research/project/academic/articles/microsoft-academic-uses-knowledge-address-problem-conflation-disambiguation/.

[5] G. J. Breugelmans, G. Roberge, C. Tippett, M. Durning, D. B. Struck and M. M. Makanga, "Scientific impact increases when researchers publish in open access and international collaboration: A bibliometric analysis on poverty-related disease papers," *PLOS One,* 2018.
