# Supplementary material for: Scientometric trends for coronaviruses and other emerging viral infections
Source: Gigascience. 2020 Aug 17;9(8):giaa085. doi: 10.1093/gigascience/giaa085 (PMC7429184; doi:10.1093/gigascience/giaa085)
Supplement: giaa085_Review_Response2 [file giaa085_review_response2.pdf]

"Scientometric Trends for Coronaviruses and Other Emerging Viral Infections"- Response to Reviewers

We thank the reviewer for the constructive comments. We really appreciate the reviewer's inspection of the code and trial run. We have addressed the reviewer's concerns and have improved the paper accordingly. We improved the READMEs to make the code usage easier. We also improved the code and explanation according to the reviewer's recommendations, and uploaded it to GigaDB.

Comment 1:
When I asked whether two significant digits are appropriate here, I was suspecting one would be most appropriate, two might perhaps be acceptable, but the authors' decision to go for five now does not make sense to me.

Response 1:
Thank you for clarifying, we changed it to one significant digit.

Comment 2:
I think SVG would be preferable over PNG and also address Reviewer 1's comment about the legibility of the figures better. However, I suspect OUP's publishing workflows cannot handle SVG properly.

Response 2:
We agree that SVG as a format has many advantages, originally, we tried adding the figure as SVG to the paper but the text inside the figures was not displayed correctly after the latex compilation. CTAN documentation recommends converting SVG to PDF using Inkscape but then it would not be web compatible.
In the current submission, we also added all the figures as an SVG, in case OUP would like to use them.

Comment 3:
If you say that you "believe the sharing of code and data is very important for driving science forward", then I have a hard time understanding why you shared the code and data the way you did, which made it very hard to review or to contribute improvements (see below).

Response 3:
We are working already for about two months on uploading the data to the Journal FTP. We had many problems with the data uploading to the journal FTP, disconnections, slow upload speeds (about 20 kb/s) corrupted files, etc. Currently, the upload speed is better than before but only at night. We already finished uploading everything without corruptions (md5 validation was performed). In the previous submission, we tried to solve these problems by uploading the data to google drive where we have unlimited storage, fast upload speeds, and also there is a CLI tool that can be used to upload the data directly from the server. We hoped that this will make the data more accessible but turned out this did not work as well as we planned. We hope this is rectified in future submissions.

Comment 4:
To begin with, why am I just presented with three large files and no guidance? Wouldn't it make sense to place a README such that it is the first thing a potential user would see and interact with?

Response 4:
We have improved the READMEs fixed the typos and added additional documentation about the datasets.

Comment 5:
Without guidance, it was hard to decide which files to examine and how, so I focused on the Jupyter notebooks and Python scripts that I could find. The notebooks have apparently not been prepared for sharing (which would involve deleting all outputs, running all cells again and comparing that to the results obtained earlier).

Response 5:
As suggested we cleared the outputs of all the notebooks.

Comment 6:
The notebooks directly in ScienceDynamics-dev/examples were quite interesting in themselves as a foundation for the work reported in the manuscript, but not directly relevant to it since they seem to have been the last run well before COVID-19 made the news. This creates additional complications, e.g. ScienceDynamics-dev/examples/Part I Creating Datasets.ipynb links to https://kddcup2016.azurewebsites.net/Data, which is now a dead link, albeit its former contents and the link chain from it can be in part recovered from the Internet Archive. From a reviewer's perspective, it would have been more useful to use something like
https://doi.org/10.5281/zenodo.2628216 (that is already public and easily accessible and permanently archived with a persistent identifier) as the basis for the computations, especially since that is already cited in the manuscript (as ref. 38 in the revision). However, I understand that this resource was not available when the manuscript was first submitted

Response 6:
The MAG version used in the paper is from https://doi.org/10.5281/zenodo.2628216. Some of the notebooks in the git related to the original study for which the library was developed, that paper used the https://kddcup2016.azurewebsites.net/Data. We changed the folder structure and added a about the folder structure in the README.

Comment 7:
ScienceDynamics-dev/requirements.txt lists multiple dependencies twice

Response 7:
Thank you for bringing this to our attention, we updated the requirements.txt accordingly

Comment 8:
The pubmed.json generated by pubmed_2_json.py as per ScienceDynamics-dev/examples/Coronavirus/README.md was empty

Response 8:
We updated the README and rewritten pubmed_2_json.py to work as a CLI tool.
