# Supplementary material for: Scientometric trends for coronaviruses and other emerging viral infections
Source: Gigascience. 2020 Aug 17;9(8):giaa085. doi: 10.1093/gigascience/giaa085 (PMC7429184; doi:10.1093/gigascience/giaa085)
Supplement: Supplementary file 19 [file giaa085_mietchen_review2_gigascience_giga_d_20_00084r1.zip › giaa085_Mietchen_Review2_GIGASCIENCE_GIGA_D_20_00084R1.odt]

Comments on GIGASCIENCE GIGA-D-20-00084R1

I appreciate the changes the authors have made in the manuscript and their fruitful responses to the reviewer comments.

I think the text of the manuscript has been much improved, and I have only very few rather minor comments, which I will base on the numbered responses to my comments from the first round of review:

- Response 6: when I asked whether two significant digits are appropriate here, I was suspecting one would be most appropriate, two might perhaps be acceptable, but the authors’ decision to go for five now does not make sense to me.

- Response 13: I think SVG would be preferable over PNG and also address Reviewer 1’s comment about the legibility of the figures better. However, I suspect OUP’s publishing workflows cannot handle SVG properly.

- Response 29: If you say that you “believe the sharing of code and data is very important for driving science forward”, then I have a hard time understanding why you shared the code and data the way you did, which made it very hard to review or to contribute improvements (see below).

- Response 30: I am looking forward to collaborate on curating geolocation and other relevant data.

- Response 32: I spent a lot of time again to try and run your code, and there was progress with respect to the first round, but overall, I found the experience rather frustrating and would have quit earlier in the process if I had not been interested so much in the data and methodology. 

  - To begin with, why am I just presented with three large files and no guidance? Wouldn’t it make sense to place a README such that it is the first thing a potential user would see and interact with? 

  - Next, a Google Drive folder is not very accessible from the command line, except via dedicated tools with further dependencies that complicate things. So I asked the journal to provide an FTP version, which worked fine for the most part but it seems that the biggest file from the repo did not get copied over properly, which I only realized after multiple days of shuffling the data around to different systems. In the end, I did not have access to a system that had (a) sufficient disk space and (b) sufficient bandwidth and (c) where I had sufficient permissions to install all the dependencies and, frankly, I do not think that is the duty of the reviewer to provide.

  - To the extent that I could access and unpack the provided files, I went through them mostly as a reader but ran the bits and pieces accessible to me. In doing so, I noticed that 

    - Without guidance, it was hard to decide which files to examine and how, so I focused on the Jupyter notebooks and Python scripts that I could find. The notebooks have apparently not been prepared for sharing (which would involve deleting all outputs, running all cells again and comparing that to the results obtained earlier).

    - The notebooks directly in ScienceDynamics-dev/examples were quite interesting in themselves as a foundation for the work reported in the manuscript, but not directly relevant to it, since they seem to have been last run well before COVID-19 made the news. This creates additional complications, e.g. ScienceDynamics-dev/examples/Part I Creating Datasets.ipynb links to [https://kddcup2016.azurewebsites.net/Data](https://kddcup2016.azurewebsites.net/Data) , which is now a dead link, albeit its former contents and the link chain from it can be in part recovered from the Internet Archive. From a reviewer’s perspective, it would have been more useful to use something like  [https://doi.org/10.5281/zenodo.2628216](https://doi.org/10.5281/zenodo.2628216) (that is already public and easily accessible and permanently archived with a persistent identifier) as the basis for the computations, especially since that is already cited in the manuscript (as ref. 38 in the revision). However, I understand that this resource was not available when the manuscript was first submitted.

    - ScienceDynamics-dev/requirements.txt lists multiple dependencies twice

    - The pubmed.json generated by pubmed_2_json.py as per ScienceDynamics-dev/examples/Coronavirus/README.md was empty

    - ScienceDynamics-master/README.md had about 1 typo per line, and the GitHub repo referenced therein is still not publicly accessible. I applaude the authors’ decision to post a preprint, and I went through the comments posted there, but when data and code are so central to the research and still essentially not available in public, then I am reminded of 

      - “An article about computational science in a scientific publication is not the scholarship itself, it is merely advertising of the scholarship. The actual scholarship is the complete software development environment and the complete set of instructions which generated the figures.” (John Claerbout, via [Buckheit &  Donoho, 1995](https://doi.org/10.1007/978-1-4612-2544-7_5))

Since the initial submission, the field of COVID-19-related research has grown very rapidly, and bibliometric studies on the matter have begun to appear, e.g. [Nowakowska et al.](https://doi.org/10.1016/j.biopha.2020.110451) from this week. Yet the methodology presented here is important and could assist with making sense of the research about this pathogen and this outbreak as well as past and future ones, potentially well beyond those covered in the study. The methodology is also largely applicable to other fields, as highlighted by the tutorial notebooks, so I am looking forward to see it being used in practice and developed and refined further, and once the associated resources are open, I shall be happy to contribute to them. 

Daniel Mietchen, 26 June 2020
